# Supplementary material for: Reassessment of growth-climate relations indicates the potential for decline across Eurasian boreal larch forests
Source: Nat Commun. 2023 Jun 8;14:3358. doi: 10.1038/s41467-023-39057-5 (PMC10250375; doi:10.1038/s41467-023-39057-5)
Supplement: Supplementary file 1 — Supplementary Information [file 41467_2023_39057_MOESM1_ESM.pdf]

Supplementary Information

Reassessment of growth-climate relations indicates the potential for decline over Eurasian boreal larch forests

Wenqing Li; Rubén D. Manzanedo; Yuan Jiang; Wenqiu Ma; Enzai Du; Shoudong Zhao; Tim Rademacher; Manyu Dong; Hui Xu; Xinyu Kang; Jun Wang; Fang Wu; Xuefeng Cui; Neil Pederson

The Supplementary Information is divided into four parts, containing Supplementary Tables 1-8, Supplementary Figures 1-21, and references.

Content

Part A Climate pattern of the study area **4**

Table 1 Climate conditions of Eurasian boreal larch forests under multiple Shared Socio-economic Pathways and during multiple time periods.

Figure 1 Climate changes across the Northern Hemisphere over 1960-2020.

Figure 2 Climate spaces occupied by Siberian larch (a) and Dahurian larch (b) during 1960-1990 and 1970-2000.

Figure 3 Projected future climate spaces occupied by the two boreal larch species under multiple Shared Socio-economic Pathways.

Part B Supplementary information for results and discussion **9**

Table 2 Analysis formulas of the climate boundaries between the positively-responding and negatively-responding populations with different identification probability thresholds (P_0).

Table 3 Statistics of the estimated logistic regression models.

Figure 4 Partial correlations of the metrics characterizing the temperature sensitivity with the local climatic conditions.

Figure 5 Scatter plots showing the relationships between the correlation-derived metrics and the local climatic conditions.

Figure 6 Scatter plots showing the relationships between the mean significant correlation coefficient and the local climatic conditions.

Figure 7 Probability distributions of climatic factors and geographical coordinates grouped by populations showing positive (red) and negative (blue) temperature sensitivity.

Figure 8 Distribution of the populations showing significant temperature sensitivity in the climate space.

Figure 9 Projected distributions of the positively-responding and negatively-responding regions under Shared Socio-economic Pathways (SSP) 1-26 and 3-70.

Figure 10 Area percentages of the negatively-responding region in species distributions identified by 0-25 GCMs under different projection scenarios.

Figure 11 Tree density across the distribution area of Eurasian boreal larch forests.

Part C Supplementary information for materials and method **18**

Table 4 Comparisons of the correlation results of single standardized growth series and individual tree chronology with temperature

Table 5 Global circulation models of climate projections under four Shared Socio-economic Pathways.

Figure 12 Comparisons of the metrics derived from the correlation results of single standardized growth series and individual tree chronology with temperature.

Figure 13 Comparison of the main results depending on different detrending method used.

Figure 14 Time spans of tree-ring samples.

Figure 15 Catalog of projection scenarios for the positively-responding and negatively-responding regions.

Part D Example of constructing temperature time series using the T-linked method 24

Table 6 Procedure of translating temperature values of L-2 °C and L-12 °C into dates in 1977 for Station A.

Table 7 Periods determined by paired temperature values of L-2 °C and L-12 °C and the corresponding T-linked temperature series for Station A.

Figure 16 Temperature value pair pool of Station A.

Figure 17 Steps of constructing temperature time series for a meteorological station using T-linked method.

Figure 18 Correlation results between the population chronology and the qualified T-linked temperature time series.

Figure 19 Positive correlation results between tree-level growth series and the qualified T-linked temperature time series.

Figure 20 Negative correlation results between tree-level growth series and the qualified T-linked temperature time series.

Figure 21 The qualified T-linked temperature series during 1960-1990 in this example.

Supplementary Table 8 References of the extracted population chronologies. **34**

References 35

Part A Climate pattern of the study area

Climate changes over 1960-2020 across the study area

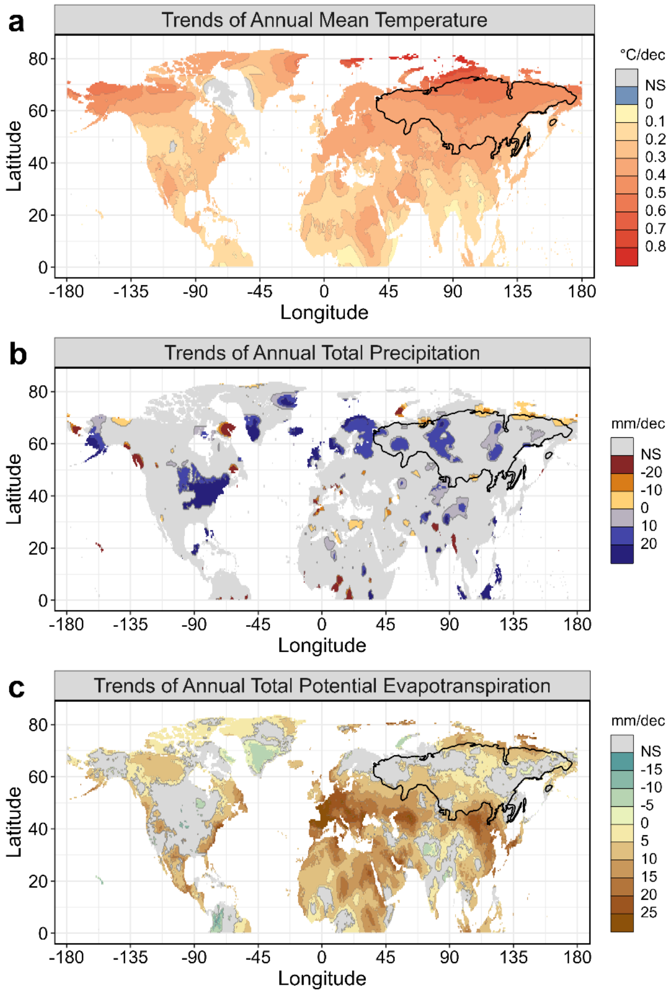

Supplementary Fig. 1| Climate changes across the Northern Hemisphere over 1960-2020. Trends of (**a**) annual mean temperature (°C dec^{-1}), (**b**) annual total precipitation (mm dec^{-1}) and (**c**) annual total potential evapotranspiration (mm dec^{-1}) over 1960-2020. The black lines denote the distribution area of Eurasian boreal larch forests. Colored cells represent significant trends (F-test, $p < 0.05$), while grey cells represent non-significant trends. Climate data are collected from CRU TS 4.05 at a spatial resolution of 0.5°.

Near current and projected climate spaces occupied by Eurasian boreal larch forests

We displayed the climate spaces occupied by Siberian larch (*Larix sibirica*) and Dahurian larch (*Larix gmelinii*) during two near current time periods (1960-1990 and 1970-2000) and four projection time periods (2021-2040, 2041-2060, 2061-2080, and 2081-2100) (Supplementary Table 1, Supplementary Figs. 2, 3). Our findings indicated that the distribution area of Siberian larch was both warmer and wetter than that of Dahurian larch (Supplementary Figs. 2, 3). During the period of 1970-2000, mean annual temperatures (MAT) of the species distribution was –2.1±3.4 °C for Siberian larch and –9.5±4.4 °C for Dahurian larch, while mean annual precipitations (MAP) were 479±125 mm and 376±126 mm, respectively (Supplementary Table 1). We also found that Eurasian boreal larch forest would experience a warmer and wetter climate in the future (Supplementary Table 1). The climate spaces occupied by the two larch species were both projected to move rightwards and upwards, i.e., to be warmer and wetter (Supplementary Fig. 3). Under the mild Shared Socio-economic Pathway (SSP) 2-45, MAT of the species distribution was projected to increase to 2.8±3.1 °C for Siberian larch and –4.3±4.0 °C for Dahurian larch, respectively, by 2100. Under the worst-case SSP5-85, MATs were projected to be 6.2±3.0 °C and –0.7±3.8 °C for the two larch species, respectively (Supplementary Table 1). MAPs would correspondingly increase to 526±141 mm for Siberian larch and 427±140 mm for Dahurian larch under SSP2-45, and 541±149 mm and 457±148 mm, respectively, under SSP5-85 (Supplementary Table 1).

Supplementary Table 1| Climate conditions of Eurasian boreal larch forests under multiple Shared Socio-economic Pathways and during multiple time periods.

Scenario	Time Period	*Larix*		*Larix sibirica*		*Larix gmelinii*	
		MAT	MAP	MAT	MAP	MAT	MAP
Baseline	1960-1990	-6.60	426	-2.35	479	-10.09	384
	1970-2000	-6.17	421	-2.12	479	-9.50	376
SSP1-26	2021-2040	-3.79	446	0.20	504	-7.08	402
	2041-2060	-3.08	454	0.88	512	-6.34	410
	2061-2080	-2.76	457	1.19	515	-6.02	413
	2081-2100	-2.82	458	1.10	515	-6.04	414
SSP2-45	2021-2040	-3.68	446	0.32	504	-6.97	402
	2041-2060	-2.56	456	1.38	513	-5.82	412
	2061-2080	-1.69	464	2.21	521	-4.89	421
	2081-2100	-1.09	469	2.76	526	-4.26	427
SSP3-70	2021-2040	-3.79	444	0.20	502	-7.07	399
	2041-2060	-2.41	455	1.52	513	-5.65	411
	2061-2080	-0.89	468	2.98	524	-4.08	426
	2081-2100	0.72	481	4.49	535	-2.39	441
SSP5-85	2021-2040	-3.50	447	0.49	504	-6.78	403
	2041-2060	-1.83	461	2.09	517	-5.05	418
	2061-2080	0.16	476	3.98	529	-2.98	436
	2081-2100	2.42	493	6.16	541	-0.66	457

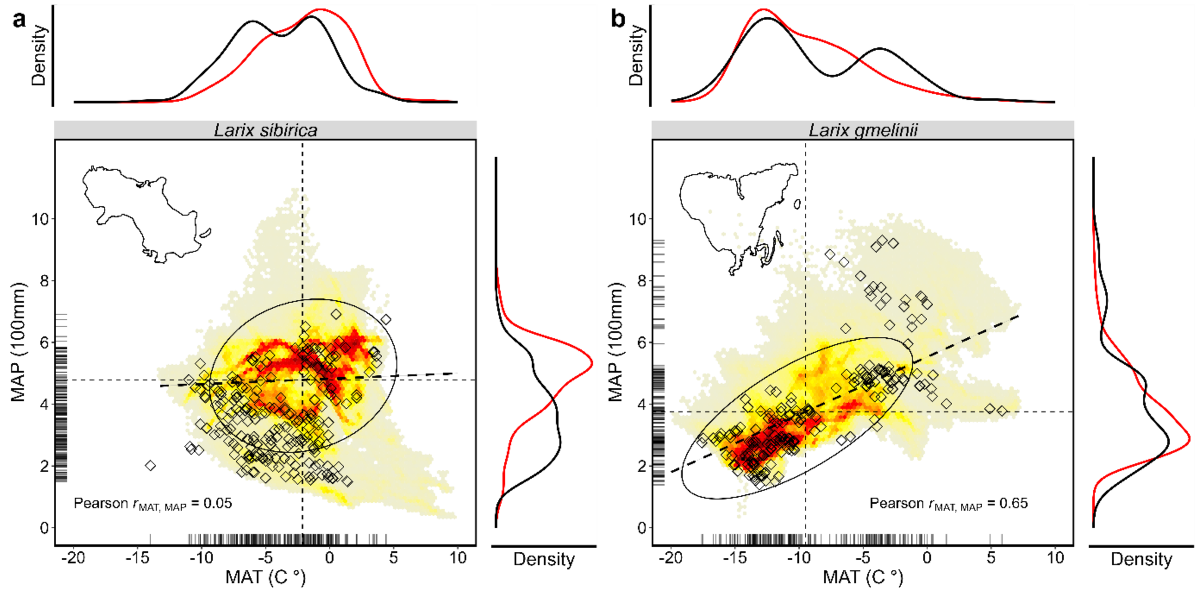

Supplementary Fig. 2| Climate spaces occupied by Siberian larch (a) and Dahurian larch (b) during 1970-2000. MAT and MAP represent mean annual temperature and precipitation, respectively. Shadows represent the climate spaces occupied by the two larch species. Gradient color represents the cell density. Straight dashed lines represent the averages of climate parameters, and slant lines represent the fitted lines between MAT and MAP using simple least square regression. Correlation coefficients (Pearson's *r*) between MAT and MAP are noted on each panel. Sampling populations were located in the climate space based on their 30-year local climate conditions. Probability distributions of the climate conditions (MAT and MAP) across the species distributions (red line) and of the sampling populations (black line) estimated by kernel density were provided. The climate datasets are collected from WorldClim at a spatial resolution of 2.5'.

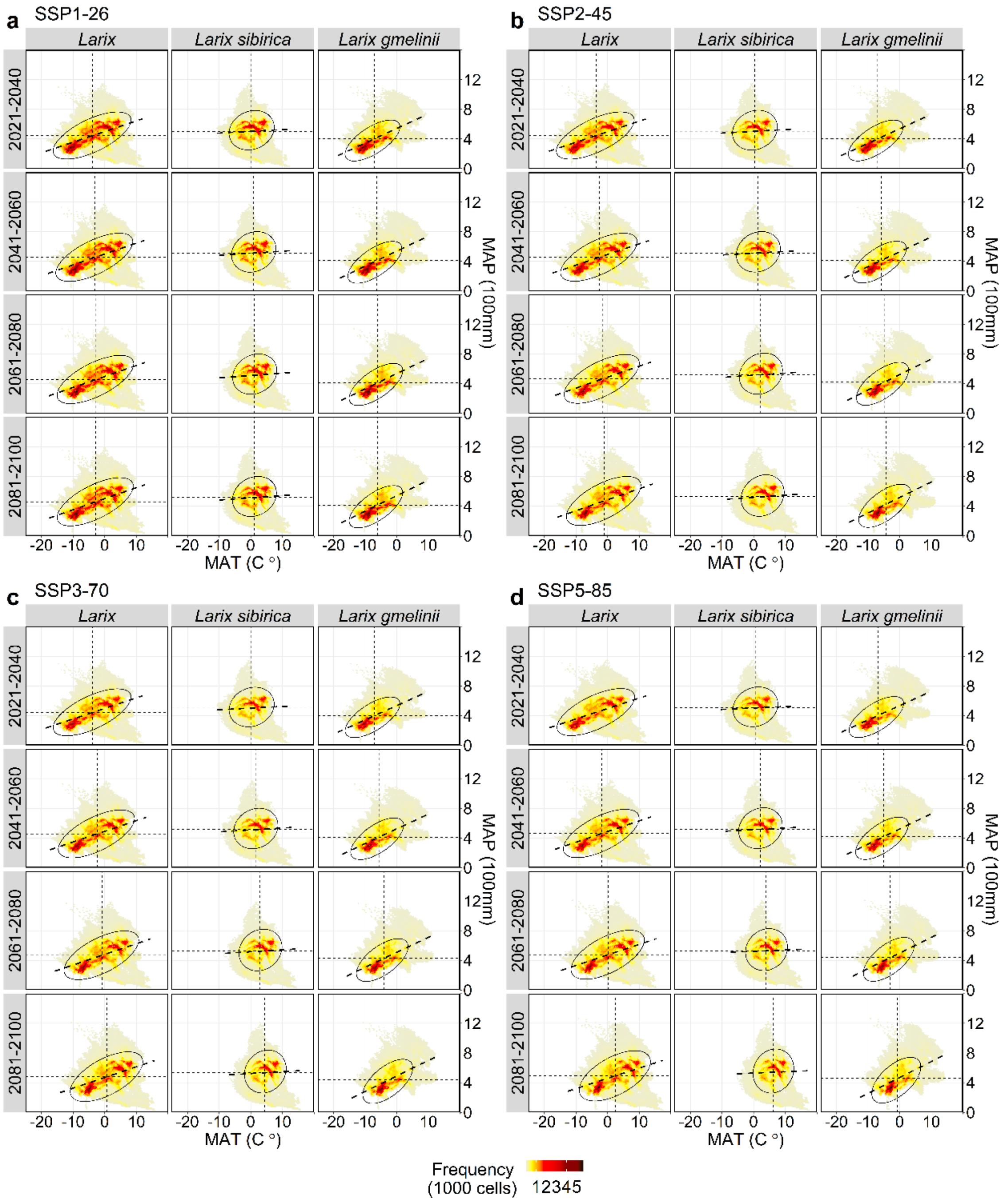

Supplementary Fig. 3| Projected future climate spaces occupied by the two boreal larch species under multiple Shared Socio-economic Pathways. a, SSP1-26; **b**, SSP2-45; **c**, SSP3-70; **d**, SSP5-85. MAT and MAP represent mean annual temperature and precipitation, respectively. Shadows represent the climate spaces occupied by the two larch species. Gradient color represents the cell density. Straight dashed lines represent the averages of climate parameters, and slant lines represent the fitted lines between MAT and MAP using simple least square regression. The climate datasets are collected from WorldClim at a spatial resolution of 2.5'. We averaged the climate projections from all the 25 GCMs under the same SSP and during the same time period to represent future climate conditions.

Part B Supplementary information for results and discussion

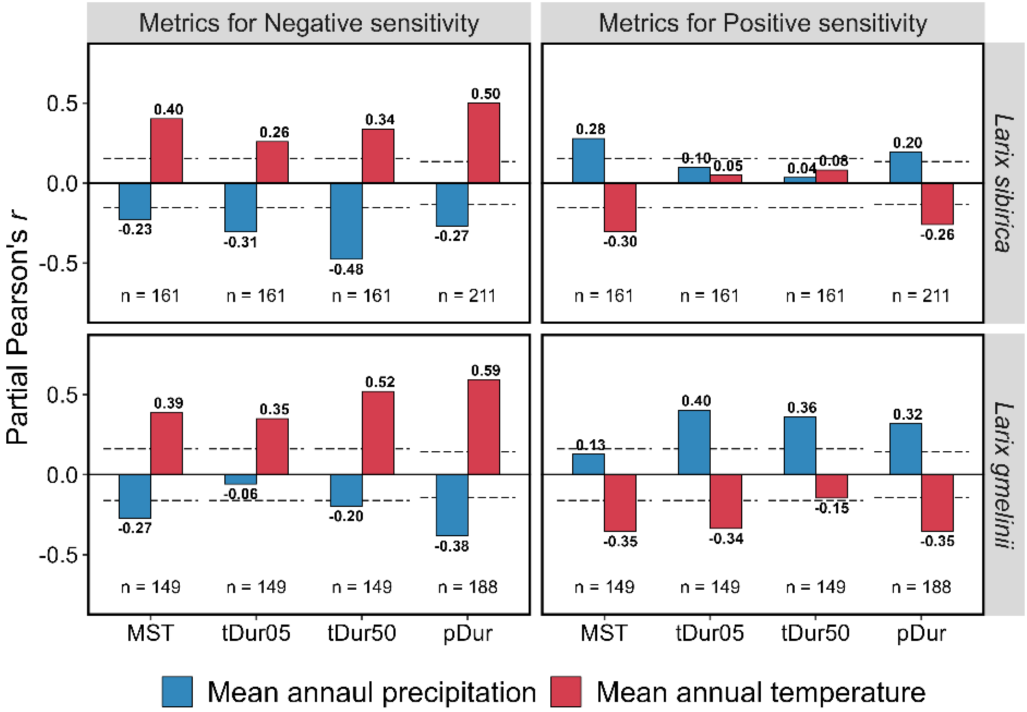

Supplementary Fig. 4| Partial correlations of the metrics characterizing the temperature sensitivity with the local climatic conditions. MST represents the maximum scope of temperature effects on populations, which is the maximum proportion of negative or positive temperature-sensitive single growth series in a population; tDur05/tDur50 represents the duration of tree-level growth-temperature response, which is the average mean number of days of the top 5%/50% T-linked temperature series in descending order of the proportion of individuals in the population that are significantly negatively or positively correlated with them; pDur represents the duration of population-level growth-temperature response, which is the average mean number of days of all T-linked temperature series with which the population chronology is significantly negatively or positively correlated. The horizontal lines denote the 0.05 significance level; red and blue represent mean annual temperature and precipitation, respectively.

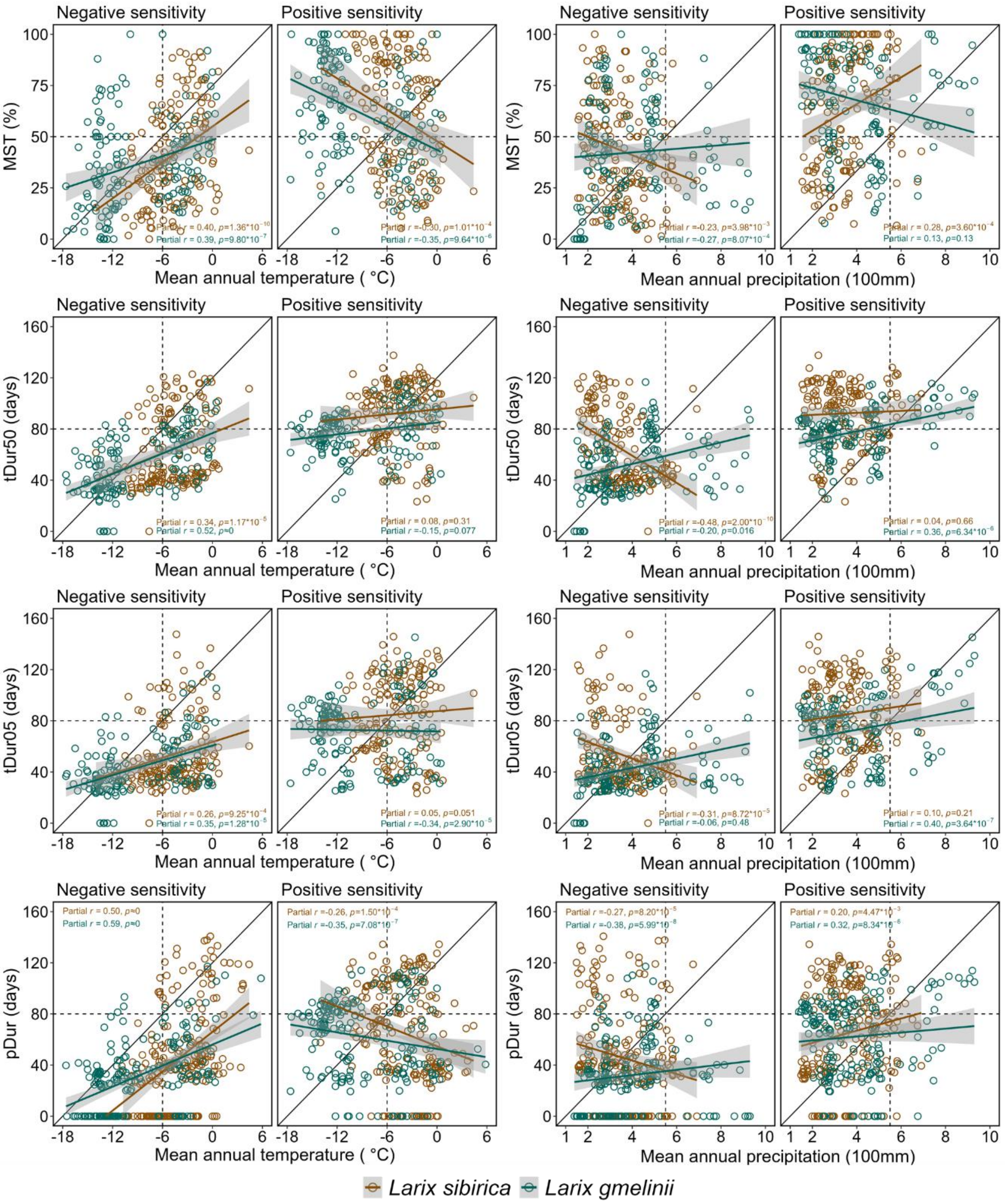

Supplementary Fig. 5| Scatter plots showing the relationships between the correlation-derived metrics and the local climatic conditions. Panels display the relationships of each correlation-derived metric with the local climatic conditions (mean annual temperature and precipitation). Orange and cyan represent Siberian larch (*n* = 161 populations for MST, tDur05 and tDur50; *n* = 211 populations for pDur) and Dahurian larch (*n* = 149 populations for MST, tDur05 and tDur50; *n* = 188 populations for pDur), respectively. Partial correlation coefficients and *p*-values (two-sided *t*-test) are noted on each panel following the same color scheme. Solid lines represent the fitted lines between metric and climatic conditions using simple least square regression, with 0.95 confidence intervals (grey shadow) around the fitted lines.

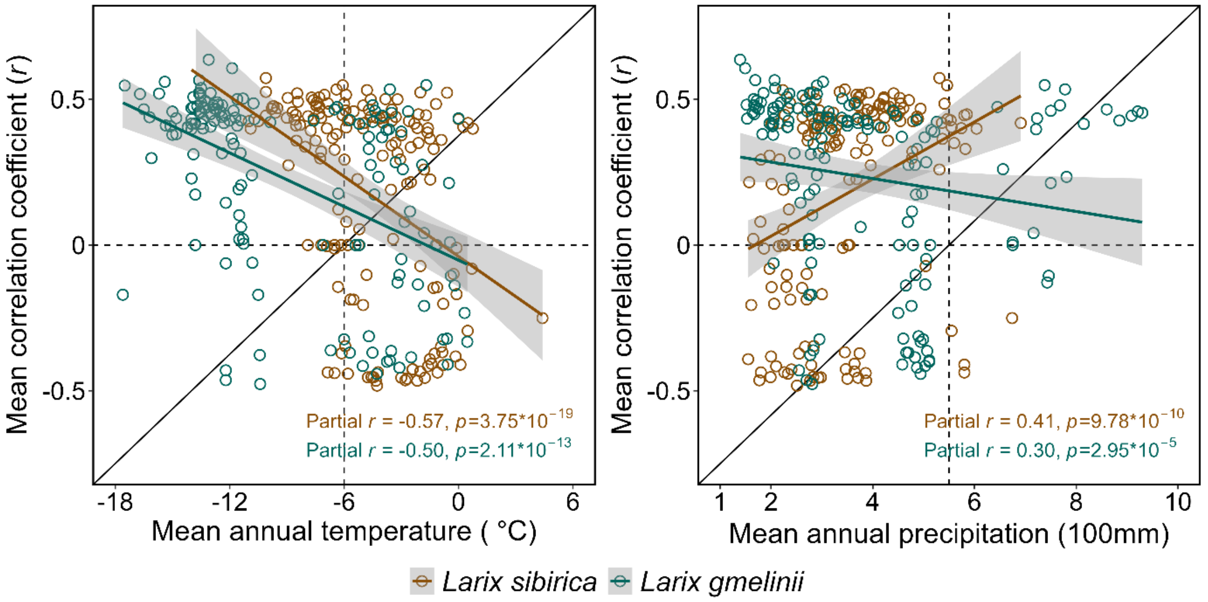

Supplementary Fig. 6| Scatter plots showing the relationships between the mean significant correlation coefficient and the local climatic conditions. Panels display the relationships of each correlation-derived metric with the local climatic conditions (mean annual temperature and precipitation). Orange and cyan represent Siberian larch (*n* = 211 populations) and Dahurian larch (*n* = 188 populations), respectively. Partial correlation coefficients and *p*-values (two-sided *t*-test) are noted on each panel following the same color scheme. Solid lines represent the fitted lines between metric and climatic conditions using simple least square regression, with 0.95 confidence intervals (grey shadow) around the fitted lines.

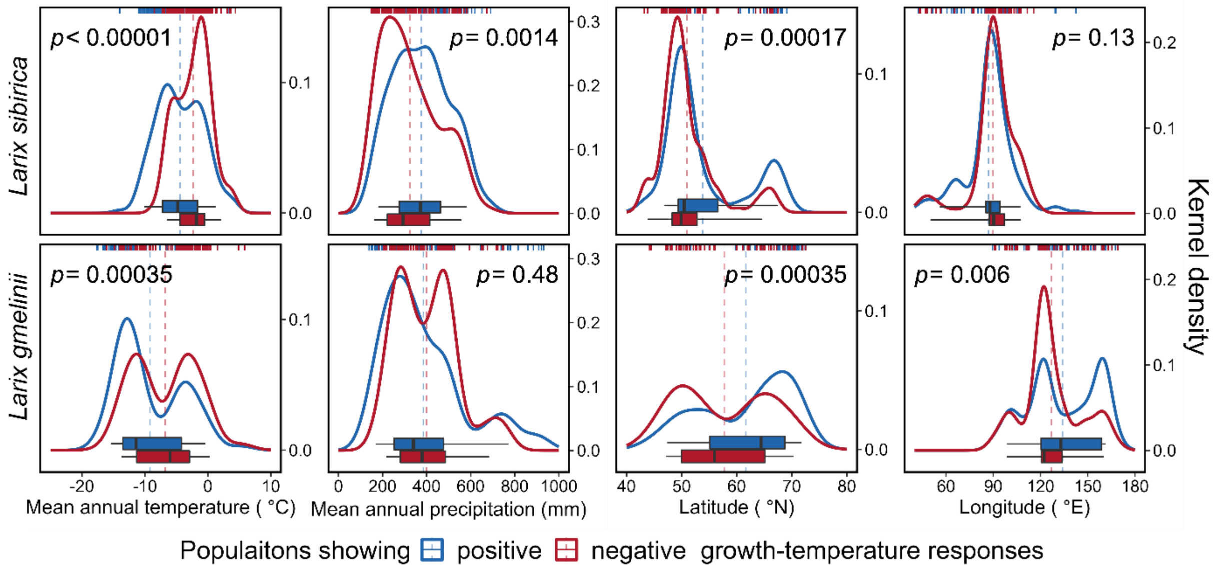

Supplementary Fig. 7| Probability distributions of climatic factors and geographical coordinates grouped by populations showing positive (red) and negative (blue) temperature sensitivity. The distribution functions were estimated by kernel density. Two-sided Student's *t*-tests are used to assess the significance in the differences between two groups (*p*-values are noted on each panel; *n* = 97 and 153 populations showing negative and positive temperature sensitivity, respectively, for Siberian larch, and *n* = 117 and 159 for Dahurian larch). Boxes represent the 25th, 50th and 75th quantiles, and whiskers extend to the 5th and 95th quantiles. Vertical dashed lines represent the average values of each factor.

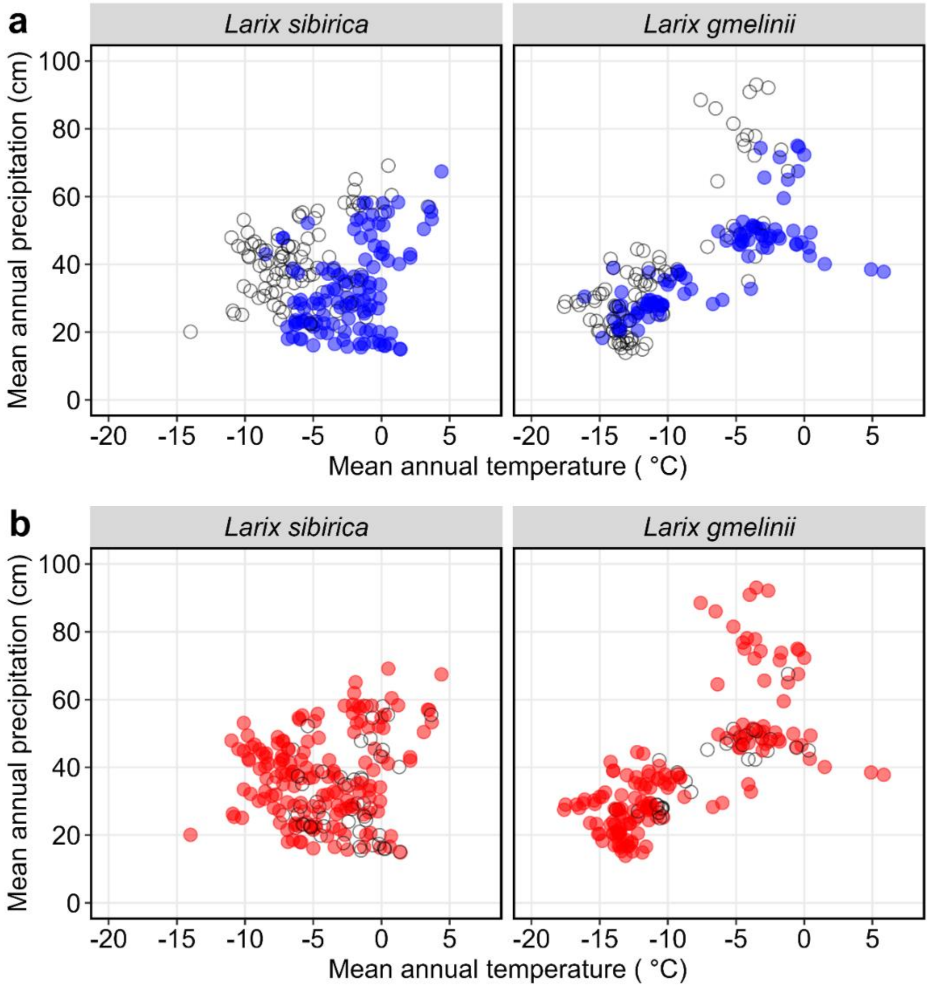

Supplementary Fig. 8| Distribution of the populations showing significant temperature sensitivity in the climate space. Plots **a** and **b** display the distributions of the populations showing significant negative (blue circles) and positive (red circles) growth responses to temperature in the climate space, respectively. Hollow circles represent the populations without corresponding temperature sensitivity.

Supplementary Table 2| Analysis formulas of the climate boundaries between the positively-responding and negatively-responding populations with different identification probability thresholds (P_0).

P_0	***Larix sibirica***	***Larix gmelinii***
0.50	MAP – 49.3×MAT – 605.3 = 0	MAP – 42.4×MAT – 810.6 = 0 (MAT > -12.5)
0.75	MAP – 50.9×MAT – 514.7 = 0	MAP – 42.4×MAT – 747.3 = 0 (MAT > -12.5)
0.95	MAP – 50.9×MAT – 362.4 = 0	MAP – 42.4×MAT – 641.1 = 0 (MAT > -12.5)

* MAT and MAP represent mean annual temperature (°C) and precipitation (mm), respectively.

Supplementary Table 3| Statistics of the estimated logistic regression models.

**Species**	**Parameter**	**Standard Error**	***z* value**	***p* value (Wald test)**
Larix sibirica	MAT (w_1)	0.085192	-7.012	2.35e-12
	MAP (w_2)	0.001976	6.132	8.65e-10
	intercept (w_3)	1.027087	-7.143	9.11e-13
Larix gmelinii	MAT (w_1)	0.154961	-4.751	2.02e-06
	MAP (w_2)	0.003725	4.664	3.11e-06
	intercept (w_3)	2.794754	-5.038	4.71e-07

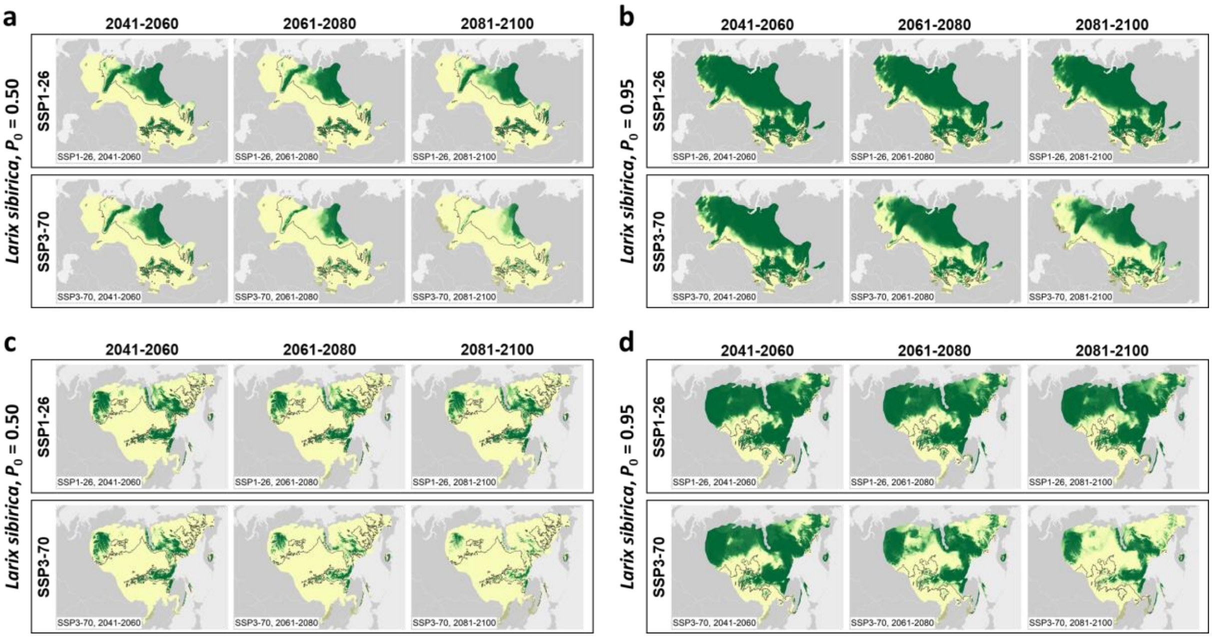

Supplementary Fig. 9| Projected distributions of the positively-responding and negatively-responding regions under Shared Socio-economic Pathways (SSP) 1-26 and 3-70. a-d. Results of Siberian larch and Dahurian larch identified by probability thresholds (P_0) of 0.50 and 0.95, respectively. Light yellow to dark green represents the decreasing proportion of the climate projections from 25 GCMs that identified the negatively-responding regions under corresponding projection scenarios; black lines represent the baseline (1970-2000) boundaries between the positively-responding and negatively-responding regions with corresponding probability thresholds. Grey shadows represent the distribution areas where projected climatic conditions falling outside the baseline climate space of boreal larch.

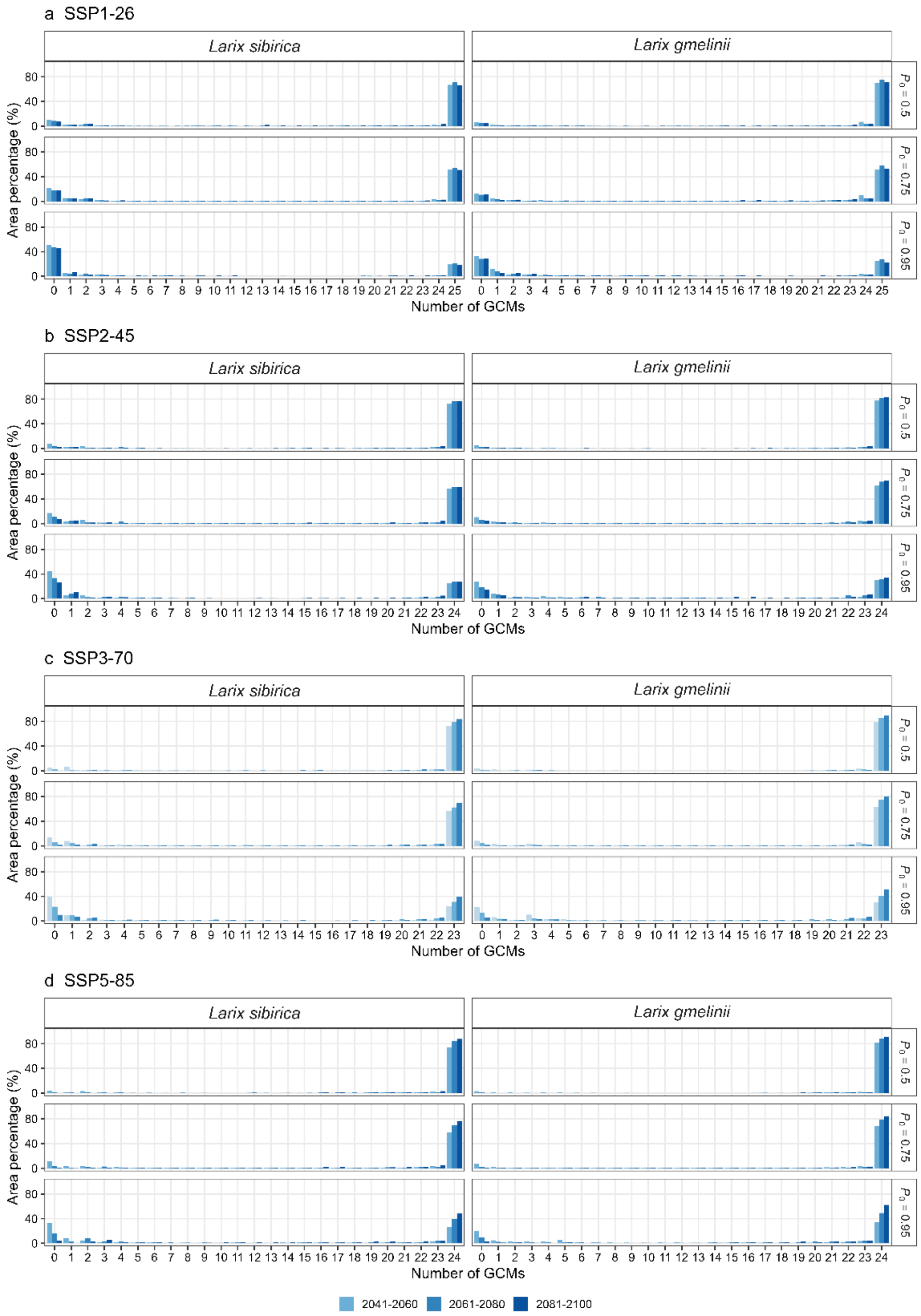

Supplementary Fig. 10| Area percentages of the negatively-responding region in species distributions identified by 0-25 GCMs under different projection scenarios. Plots **a-d** display the results under Shared Socio-economic Pathway (SSP) 1-26, 2-45, 3-70, and SSP5-85, respectively. The plot is paneled by species and identification probability thresholds (P_0); colors represent projection time periods of 2041-2060, 2061-2080 and 2081-2100.

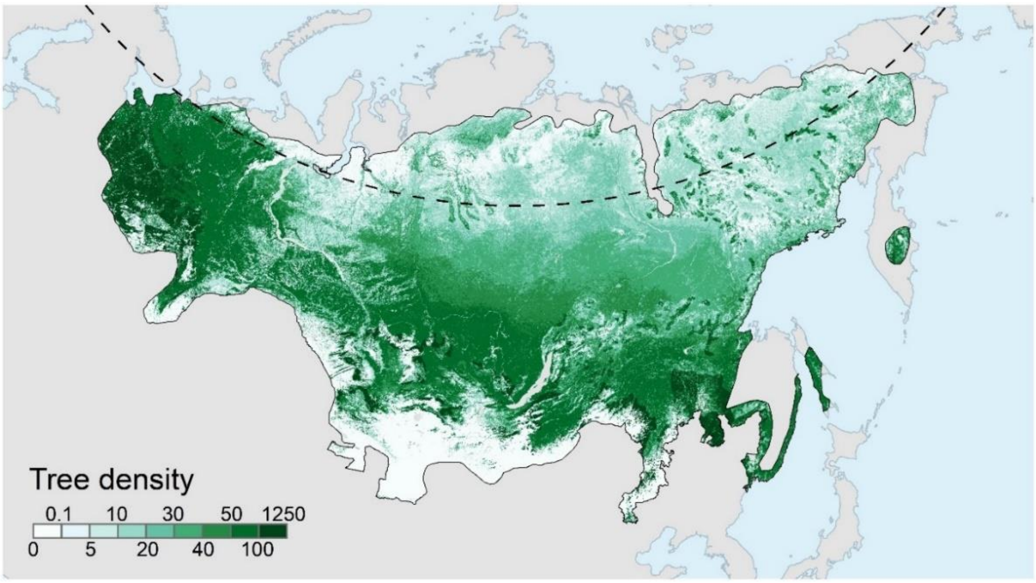

Supplementary Fig. 11| Tree density across the distribution area of Eurasian boreal larch forests. Tree density at the 1-km^2 pixel scale [1].

Part C Supplementary information for materials and methods

Supplementary Table 4| Comparisons of the correlation results of single standardized growth series and individual tree chronology with temperature

Species	Time period	Total number	Method*	Sensitive pct. (%)	Negative sensitive pct. (%)	Positive sensitive pct. (%)
**Single standardized growth series**						
Larix sibirica	1960-1990	2636	TL	2548 (96.7%)	1448 (54.9%)	2312 (87.7%)
			CB	2047 (77.7%)	714 (27.1%)	1675 (63.5%)
			CT	2028 (76.9%)	610 (23.1%)	1641 (62.3%)
	1970-2000	2053	TL	1982 (96.5%)	1432 (69.8%)	1568 (76.4%)
			CB	1424 (69.4%)	809 (39.4%)	835 (40.7%)
			CT	1427 (69.5%)	806 (39.3%)	759 (37.0%)
Larix gmelinii	1960-1990	3508	TL	3361 (95.8%)	2073 (59.1%)	3012 (85.9%)
			CB	2547 (72.6%)	1089 (31.0%)	2082 (59.4%)
			CT	2666 (76.0%)	864 (24.6%)	2189 (62.4%)
	1970-2000	2130	TL	2084 (97.8%)	1434 (67.3%)	1829 (85.9%)
			CB	1393 (65.4%)	566 (26.6%)	1090 (51.2%)
			CT	1575 (73.9%)	699 (32.8%)	1107 (52.0%)
**Individual tree chronology**						
Larix sibirica	1960-1990	1823	TL	1754 (96.2%)	972 (53.3%)	1610 (88.3%)
			CB	1451 (79.6%)	482 (26.4%)	1214 (66.6%)
			CT	1444 (79.2%)	393 (21.6%)	1202 (65.9%)
	1970-2000	1452	TL	1399 (96.3%)	948 (65.3%)	1150 (79.2%)
			CB	1040 (71.6%)	559 (38.5%)	666 (45.9%)
			CT	1033 (71.1%)	529 (36.4%)	609 (41.9%)
Larix gmelinii	1960-1990	1815	TL	1759 (96.9%)	1033 (55.8%)	1597 (88.0%)
			CB	1394 (76.8%)	582 (32.1%)	1181 (65.1%)
			CT	1448 (79.8%)	429 (23.6%)	1225 (67.5%)
	1970-2000	994	TL	976 (98.2%)	617 (62.1%)	863 (86.8%)
			CB	679 (68.3%)	272 (27.4%)	563 (56.6%)
			CT	772 (77.7%)	304 (30.6%)	591 (59.5%)

* TL: T-linked method; CB: moving calendar-based method; CT: calendar-based T-linked method.

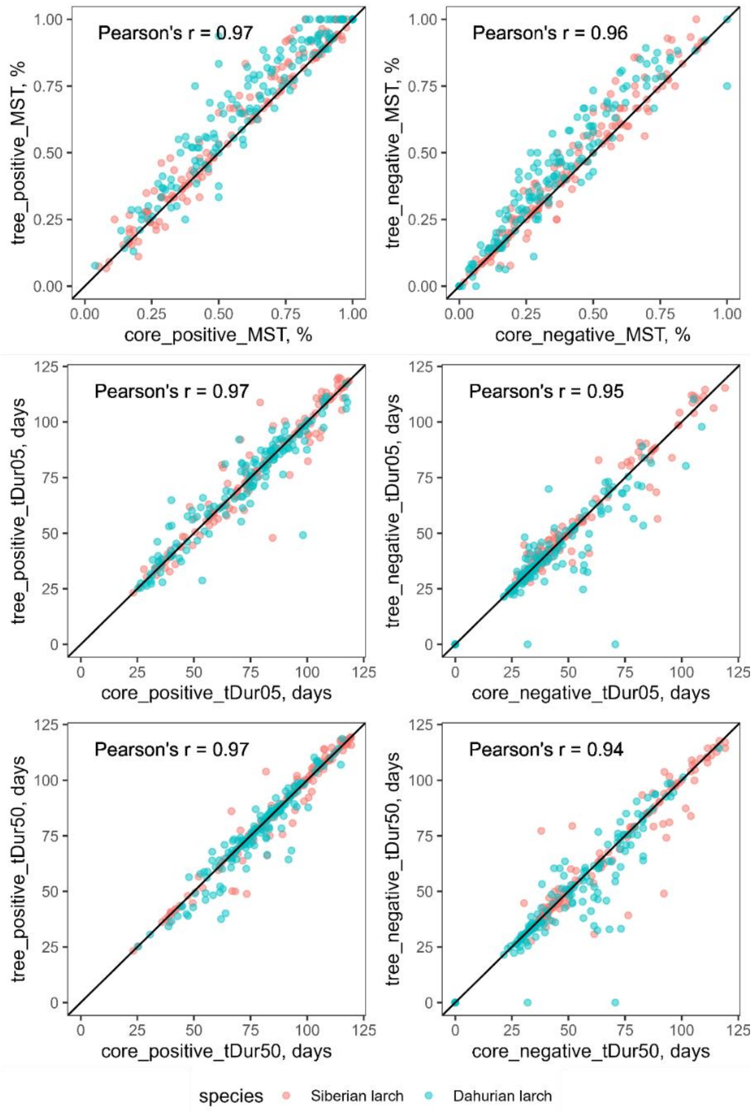

Supplementary Fig. 12| Comparisons of the metrics derived from the correlation results of single standardized growth series and individual tree chronology with temperature. The plot is paneled by the correlation-derived metrics (MST, tDur05, and tDur50 in rows) and the temperature sensitivity signs (negative and positive in columns). In each panel, the *x-axis* and *y-axis* represent the metrics calculated based on single standardized growth series (core) and individual tree chronology (tree), respectively, and the Pearson correlation coefficient between them is noted on each panel. Red and blue points represent sampling populations of Siberian larch and Dahurian larch, respectively. MST represents the maximum scope of temperature effects on populations, which is described by the maximum proportion of negative or positive temperature-sensitive single growth series or individual tree chronologies in a population. tDur05/tDur50 represents the duration of tree-level growth-temperature response, which is described by the average mean number of days of the top 5%/50% T-linked temperature series in descending order of the proportion of single growth series or individual tree chronologies in the population that are significantly negatively or positively correlated with them.

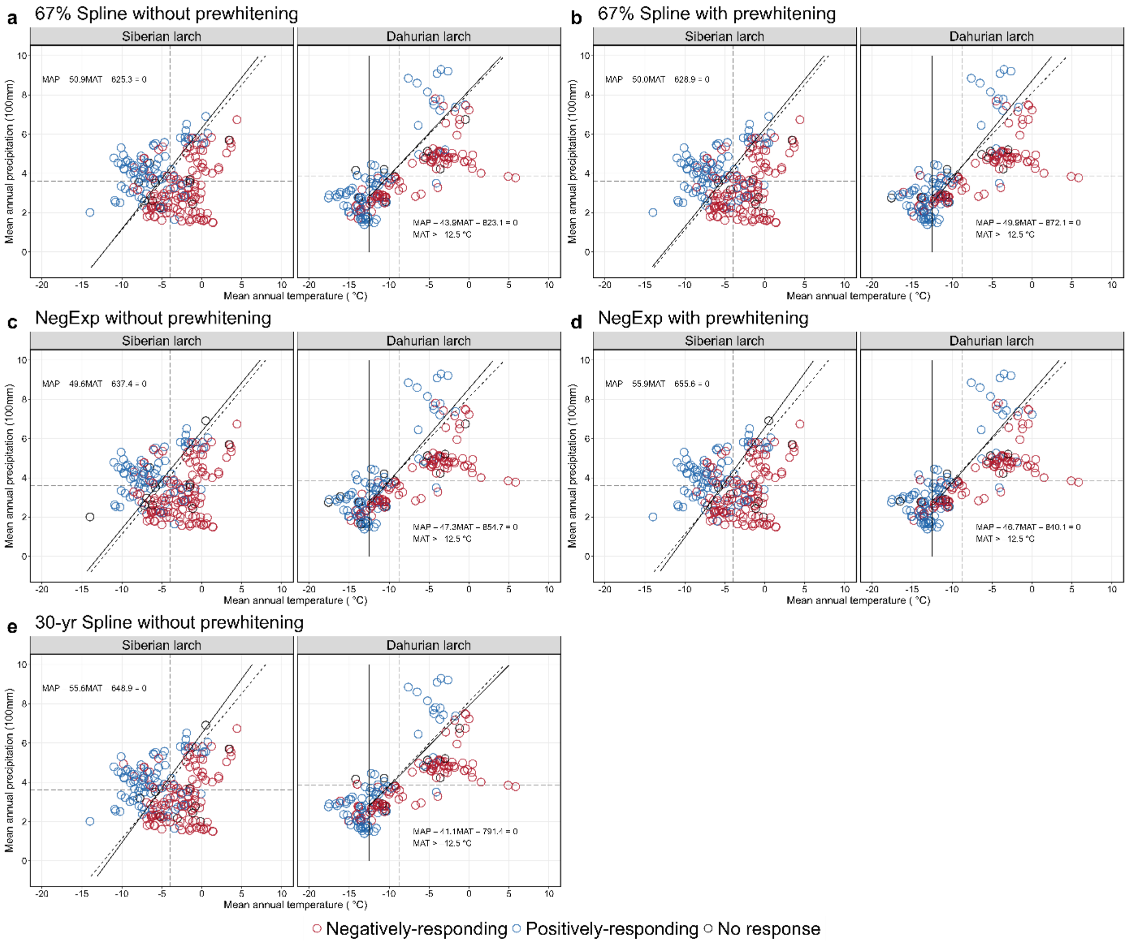

Supplementary Fig. 13| Comparison of the main results depending on different detrending method used. We calculated the main results used the growth series detrended by different methods, with and without prewhitening. **a.** 67% length spline function without prewhitening; **b.** 67% length spline function with prewhitening; **c.** negative exponential function without prewhitening; **d.** negative exponential function with prewhitening; **e.** 30-year length spline function without prewhitening. In each panel, the horizontal and vertical dashed lines denote the average values of climatic conditions, the slant dashed line represents the 0.50-probability climate boundary estimated based on the growth series detrended by 30-year length spline function with prewhitening, the slant solid line represents the 0.50-probability climate boundaries estimated based on the growth series detrended by the method labeled. The analysis formulas of the climate boundaries are noted on each panel.

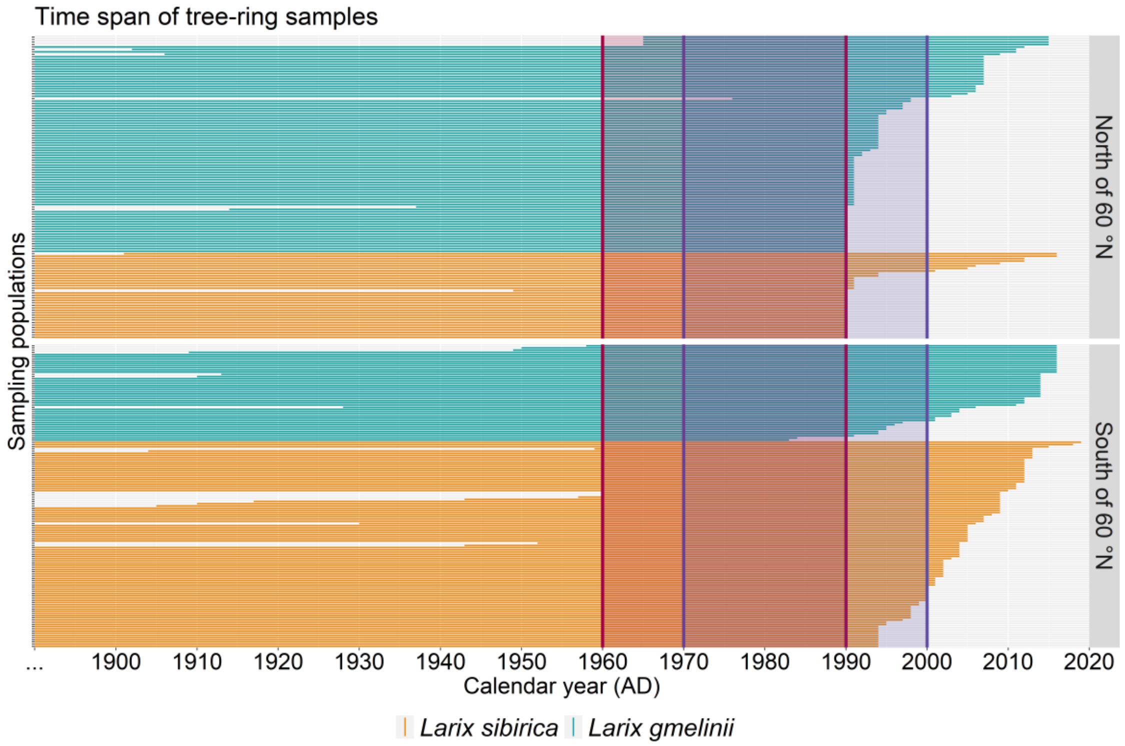

Supplementary Fig. 14| Time span of tree-ring samples. The horizontal lines correspond to the time spans of tree-ring samples of each sampling population. Orange and cyan represent Siberian larch and Dahurian larch, respectively. Sampling populations are grouped by 60°N. The analysis time periods of 1960-1990 and 1970-2000 were bounded by red and purple shadows, respectively.

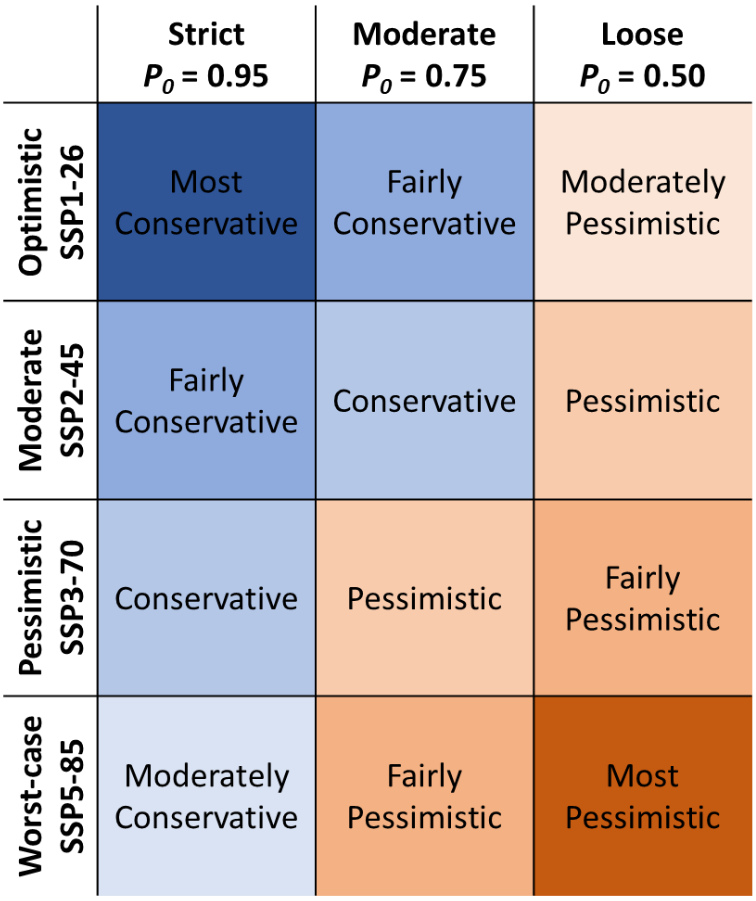

Supplementary Fig. 15| Catalog of projection scenarios for the positively-responding and negatively-responding regions. The cross-combination of the three probability thresholds (P_0 = 0.50, 0.75, and 0.95) and the four Shared Socio-economic Pathways (SSP1-26, SPP2-45, SSP3-70, and SSP5-85) constructs the gradient projection scenarios, from the most conservative scenario (P_0 = 0.95 and SSP1-26) to the most pessimistic scenario (P_0 = 0.50 and SSP5-85). The looser the probability threshold, the worse the SSP, and the more pessimistic the estimation, otherwise, the reverse.

Supplementary Table 5| Global circulation models of climate projections under four Shared Socio-economic Pathways.

Global Circulation Model	ssp126	ssp245	ssp370	ssp585
ACCESS-CM2	●	●	●	●
ACCESS-ESM1-5	●	●	●	●
BCC-CSM2-MR	●	●	●	●
CanESM5	●	●	●	●
CanESM5-CanOE	●	●	●	●
CMCC-ESM2	●	●	●	●
CNRM-CM6-1	●	●	●	●
CNRM-CM6-1-HR	●	●	●	●
CNRM-ESM2-1	●	●	●	●
EC-Earth3-Veg	●	●	●	●
EC-Earth3-Veg-LR	●	●	●	●
FIO-ESM-2-0	●	●	○	●
GFDL-ESM4	●	○	●	○
GISS-E2-1-G	●	●	●	●
GISS-E2-1-H	●	●	●	●
HadGEM3-GC31-LL	●	●	○	●
INM-CM4-8	●	●	●	●
INM-CM5-0	●	●	●	●
IPSL-CM6A-LR	●	●	●	●
MIROC-ES2L	●	●	●	●
MIROC6	●	●	●	●
MPI-ESM1-2-HR	●	●	●	●
MPI-ESM1-2-LR	●	●	●	●
MRI-ESM2-0	●	●	●	●
UKESM1-0-LL	●	●	●	●
Total	25	24	23	24

Part D Example of constructing temperature time series using the T-linked method

Variation in tree growth is usually related to climatic variation. However, identifying the climate factors and time periods over which climate affects tree growth has been problematic [2]. General methods for detecting growth-temperature responses typically correlate annual tree growth with temperature series for rigid calendar periods (months, seasons, and/or annual), meaning that choice of time periods is fixed, arbitrary, and might not follow what the physiology of trees require for growth. For forests under continuous climatic stress or in regions with consistent and long growing seasons, correlating annual growth against monthly or seasonal temperature series is relatively effective to investigate growth-temperature relations. However, for forests with comparably short and variable growth-temperature response windows, it is not strictly appropriate to conclude that this forest is insensitive to temperature solely based on the result that tree growth does not show significant responses to temperature series for rigid calendar periods. Therefore, there is a need for a more physiologically-informed and effective method of constructing temperature series through which we can better capture growth-temperature response windows.

In attempts to capture the comparably short and variable growth-temperature response windows more effectively in boreal regions and to avoid the inappropriate assumption that the same tree growth phase is expressed during the same time periods in different years, we constructed temperature time series over the periods that are linked to intra-annual temperature variability rather than the calendar-linked periods for correlation analysis with growth series. The main innovation of this method is to use the dates that are anchored to certain temperature values, namely stably passing these values, to determine the periods for constructing temperature series, instead of the common practice of simply using calendar periods. We consequently named this method as ‘temperature-linked’ or ‘T-linked’ method. Five consecutive days is a widely-used validated threshold in meteorological research to determine a stable climate stage or process [3]. Therefore, in the T-linked method, the temperature values are translated into dates following the five-day pass rule. For each

temperature value, the last day of the first five consecutive days in a year with daily mean temperatures all above this value is identified as its anchoring date for that year. Following this rule, we can use two independent temperature values to determine a period in each year using their anchoring dates in that year as the start and end points, respectively, and then a temperature series can be constructed by averaging the daily mean temperatures over these T-linked periods. The date translated from the same temperature value varies from year to year, as does the period determined by the same pair of temperature values. This flexibility implies that the correlation window also varies from year to year, in ways reflecting the great inter-annual variations in temperature across boreal regions that trees experience. By independently shifting the paired two temperature values in steps of 0.5°C, a batch of T-linked temperature series can be constructed for each meteorological station. To elaborate on this method, we provide an example of constructing the T-linked temperature series for the sample meteorological Station A during 1960-1990.

The first step is to determine the domain of temperature value for Station A. The determination of this domain should ensure coverage of the entire growing season. Both the NDVI-derived growing seasons and the VSL-modelled tree growth data suggest that the thermal limits of the start and end of the growing season in boreal Eurasia can both reach down to 0°C [4]. Furthermore, the intra-annual temperature variability is roughly symmetrical in the continental climate zone. Therefore, to ensure coverage throughout the growing season, the temperature values were selected from a 0.5-interval domain that starts from 0°C to mean annual maximum daily mean temperature record of the meteorological station and then ends down back to 0°C. The values on the left and right sides of the symmetric intra-annual temperature variability are distinguished by labels of ‘L’ and ‘R’. Mean annual maximum temperature during 1960-1990 in Station A is 23.9°C, as so the selection of temperature value starts from 0°C and increases at a step of 0.5 °C up to 24.0°C and then decreases back down to 0°C at the same steps, forming the domain of [L-0, L-0.5, L-1.0, L-1.5, …, L-22.5, L-23.0, L-23.5, L-24.0, R-24.0, R-23.5, R-23.0, R-22.5, …, R-1.5, R-1.0, R-0.5, R-0]. The translations for left and right temperature values are conducted on the left and right sides of the daily temperature

curve, respectively.

Next, any two values within this domain can be paired to determine a T-linked period in each year, and then to construct a T-linked temperature series with varying mean lengths (in the number of days, Supplementary Fig. 16). It should be noted, however, that some of the higher temperature values can only be successfully translated into dates in the relatively warm years. That is to say, these high values exist only to ensure coverage throughout the growing season, and ultimately do not participate in the construction of temperature series (Supplementary Fig. 16). We introduce this step by using the temperature value pair of L-2°C and L-12°C to construct a T-linked temperature series. The translation process from temperature value to dates is showed in Supplementary Table 6 through the examples of translating these two values to dates in 1977. The counting procedure for left temperature value starts from January 1st in this study, while the counting for right temperature value starts from the last day of each year. As shown in Supplementary Table 6, non-consecutive days above 2°C or 10°C were not considered to start the counting. If the daily mean temperature drops below 2°C on the fifth day following four consecutive days at or above 2°C, we should restart the counting from the next day with mean temperature at or above 2°C (Supplementary Table 6). We continue this counting for a temperature value until the first time the daily mean temperatures is equal to or above it occurs for five consecutive days. As shown, in 1977, the date linked to L-2°C is April 21th, while the date linked to L-12°C is June 5th. Which is to say, the T-linked period determined by temperature value pair of L-2°C and L-12°C in 1977 is from April 21th to June 5th (Supplementary Table 6, Supplementary Fig. 17a). This period is 46 days long and the daily mean temperatures during this period are averaged (7.98°C) as one value of the T-linked temperature series determined by L-2°C and L-12°C (Supplementary Table 7). The same counting procedures were conducted year by year to complete this temperature series (Supplementary Table 7, Supplementary Fig. 17b).

The same process described above was performed for each pair of temperature values within the domain, so that we obtained a batch of T-linked temperature series for Station A (Supplementary Figs. 17c, 21). For each of these temperature series, the years in which either

of the paired two temperature values was too high to be translated into a date or the T-linked periods were shorter than 10 days were excluded from the series. After being filtered, the T-linked temperature series longer than 25 years during 1960-1990 were considered as qualified (colored tiles in Supplementary Fig. 16) for subsequent growth-temperature correlation analyses.

Then, both the population- and tree-level growth series were correlated with all these qualified T-linked temperature series one by one. Population-level correlation results intuitively showed where significant growth-temperature responses occur (Supplementary Fig. 18). On top of this, tree-level correlation results further displayed the proportion of tree individuals in this population that were positively and negatively correlated with each T-linked temperature series (Supplementary Figs. 19, 20).

Supplementary Table 6| Procedure of translating temperature values of L-2 °C and L-12 °C into dates in 1977 for Station A

Temperature value of L-2°C														
**Month**							**April**							
**Date**	...	10th	11th	12th	13th	14th	15th	16th	17th	18th	19th	20th	21th	22th
**T_{mean} (°C)**	...	0.6	2.8	3.9	5.6	3.9	-2.8	0.0	5.0	7.8	10.0	8.9	5.0	-1.1
**Count**	...	**0**	**1**	**2**	**3**	**4**	**0**	**0**	**1**	**2**	**3**	**4**	**5**	**-**
	...	×	×	×	×	×	×	×	×	×	×	×	√	-
Temperature value of L-12°C														
**Month**							**June**							
**Date**	...	26th	27th	28th	29th	30th	31st	1th	2nd	3rd	4th	5th	6th	7th
**T_{mean} (°C)**	...	13.9	13.3	11.1	9.4	10.0	11.7	14.4	17.2	16.7	19.4	16.7	12.8	13.3
**Count**	...	**1**	**2**	**0**	**0**	**0**	**0**	**1**	**2**	**3**	**4**	**5**	**-**	**-**
	...	×	×	×	×	×	×	×	×	×	×	**√**	-	-

Supplementary Table 7| Periods determined by paired temperature values of L-2 °C and L-12 °C and the corresponding T-linked temperature series for Station A

**Year**	...	**1974**	**1975**	**1976**	**1977**	**1978**	**1979**	**1980**	**1981**	**1982**	**1983**
L-2 °C	...	04/20	05/06	04/28	04/21	04/25	04/21	05/01	04/15	04/15	05/03
L-12 °C	...	05/24	06/01	06/12	06/05	06/04	05/20	05/20	05/19	05/26	05/19
Length	...	35d	27d	46d	46d	41d	30d	20d	35d	42d	17d
Mean	...	8.4°C	10.0°C	10.2°C	8.0°C	10.3°C	7.0°C	10.0°C	9.1°C	9.1°C	10.6°C

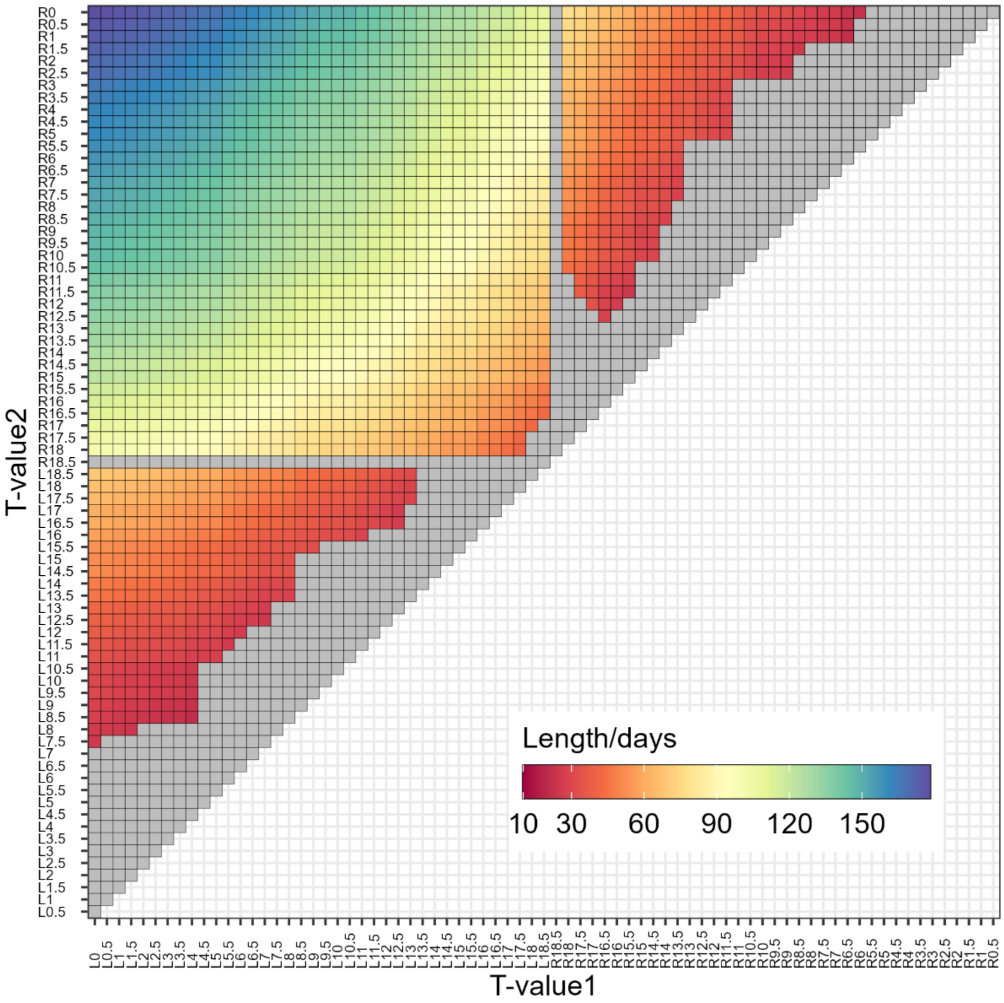

Supplementary Fig. 16| Temperature value pair pool of Station A. Coordinate axes of this plot are the domain of temperature value for Station A. Each tile in the plot represents a temperature value pair. Colored tiles represent the qualified T-linked temperature series, and color gradient represents the mean lengths (in the number of days) of these series. Grey tiles represent unqualified T-linked temperature series.

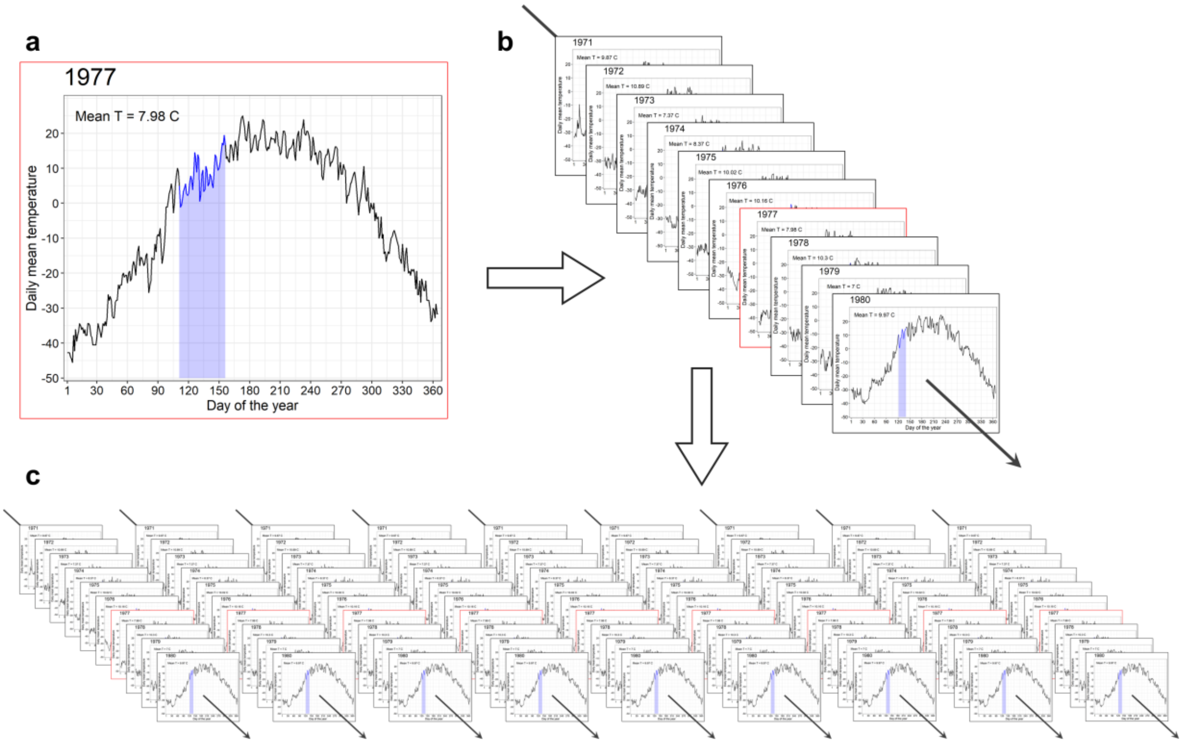

Supplementary Fig. 17| Steps of constructing temperature time series for a meteorological station using T-linked method. a. Intra-annual temperature variability of the example year 1977 for Station A. Blue section represents the T-linked period determined by the temperature value pair of L-2°C and L-12°C. **b**. Construction of the T-linked temperature series determined by the temperature value pair of L-2°C and L-12°C for Station A. **c**. Construction of the T-linked temperature series determined by other temperature value pairs for Station A.

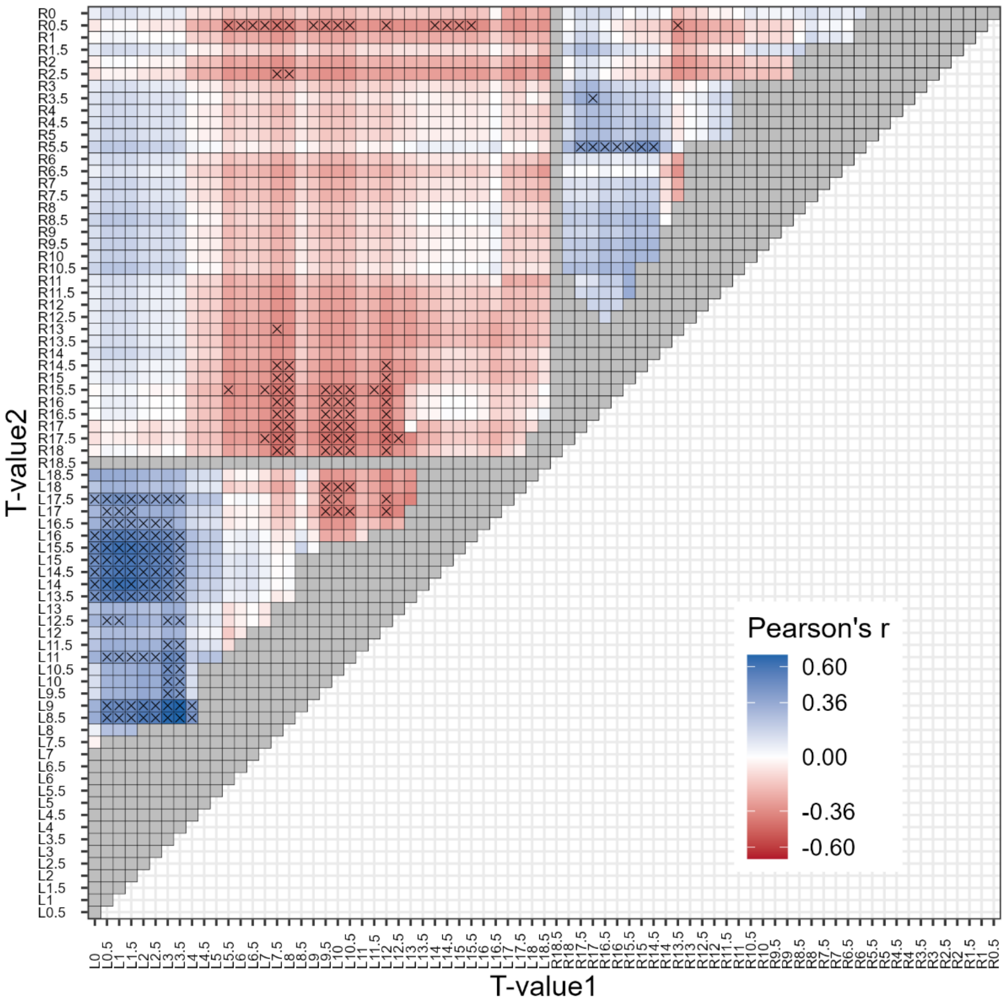

Supplementary Fig. 18| Correlation results between the population chronology and the qualified T-linked temperature time series. The blue-white-red gradient represents correlation coefficients from positive to zero to negative. Label of '×' denotes significant correlations ($p < 0.05$).

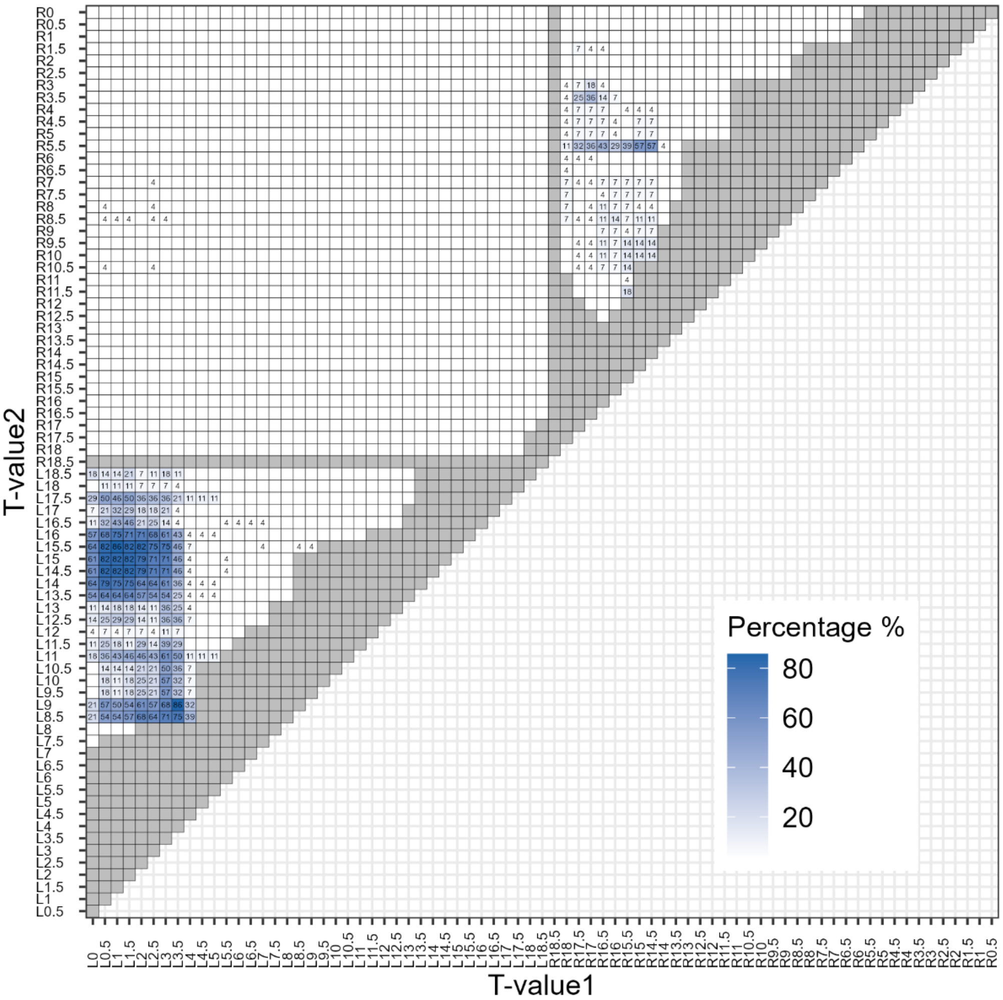

Supplementary Fig. 19| Positive correlation results between tree-level growth series and the qualified T-linked temperature time series. The proportion of individual tree-ring chronology in the population showing significantly positive correlations with each T-linked temperature series is labeled on each tile in the plot.

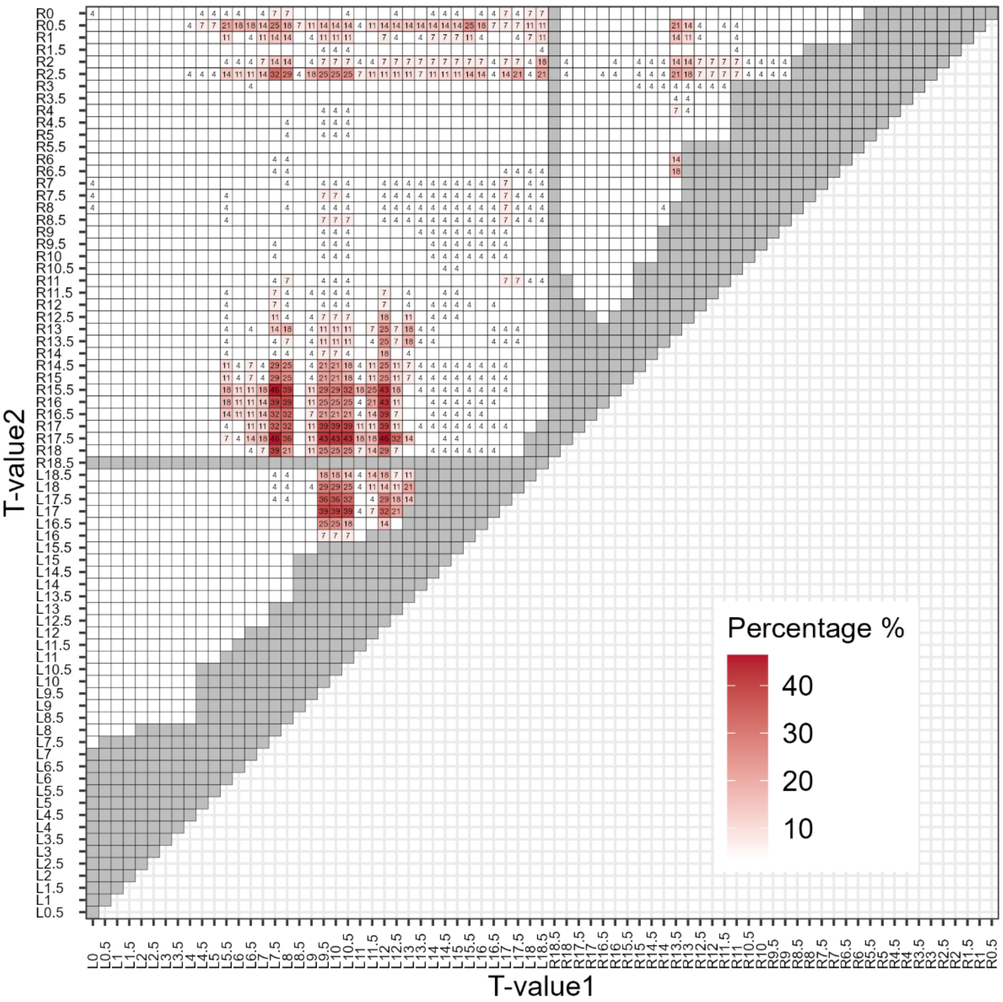

Supplementary Fig. 20| Negative correlation results between tree-level growth series and the qualified T-linked temperature time series. The proportion of individual tree-ring chronology in the population showing significantly negative correlations with each T-linked temperature series is labeled on each tile in the plot.

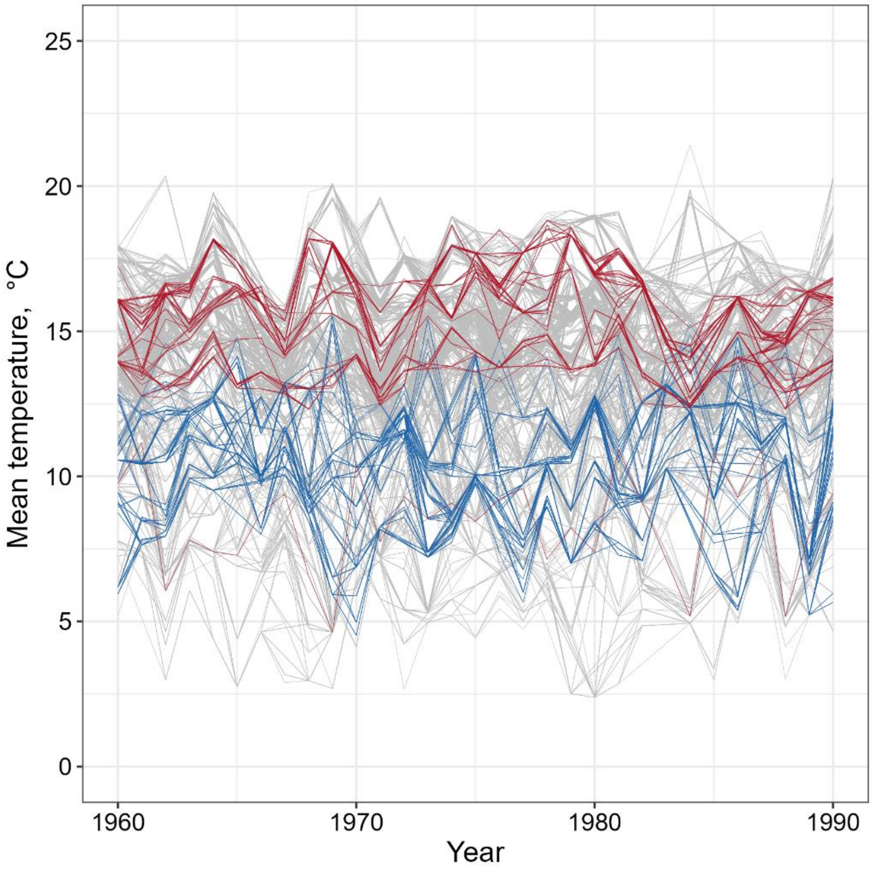

Supplementary Fig. 21| The qualified T-linked temperature series during 1960-1990 in this example. Red, blue, and grey represent the T-linked temperature series with which the population chronology was significantly negatively correlated, significantly positively correlated, and uncorrelated relationships, respectively.

Supplementary Table 8 | References of the extracted population chronologies.

ID	Site Name	Source	References
L1	WULASI	literature	Wu, et al. [5]
L2	WUFD	literature	Wu, et al. [6]
L3	WUFZ	literature	Wu, et al. [6]
L4	WUQD	literature	Wu, et al. [6]
L5	WUQZ	literature	Wu, et al. [6]
L6	WUCHE	literature	Wu, et al. [6]
L7	WUKUP	literature	Wu, et al. [6]
L8	WUSHE	literature	Wu, et al. [6]
L9	WUCEM	literature	Wu, et al. [6]
L10	SPLS	literature	Tei, et al. [7]
L11	KAZ	literature	Belokopytova, et al. [8]
L12	BID1	literature	Babushkina, et al. [9]
L13	BID2	literature	Babushkina, et al. [10]
L14	CS3	literature	Knorre, et al. [11]
L15	DSBE	literature	Babushkina and Belokopytova [12]
L16	TSLS	literature	Chen, et al. [13]
L17	AL1	literature	Gradel, et al. [14]
L18	KM1	literature	Khansaritoreh, et al. [15]
L19	BA1	literature	Dulamsuren, et al. [16]
L20	BAI	literature	Kukarskih, et al. [17]
L21	SLA	literature	Kukarskih, et al. [17]
L22	KOK	literature	Kukarskih, et al. [17]
L23	PGUF	literature	Demakov [18]
L24	KMFE	literature	Demakov [18]
L25	BKLS	literature	Khishigjargal, et al. [19]
L26	SPLG	literature	Nikolaev, et al. [20]
L27	CHUR	literature	Nikolaev, et al. [20]
L28	TYNG	literature	Nikolaev, et al. [20]
L29	CY1	literature	Arzac, et al. [21]
L30	CY2	literature	Arzac, et al. [21]
L31	CY3	literature	Arzac, et al. [21]
L32	CY4	literature	Arzac, et al. [21]
L33	CS2	literature	Kharuk, et al. [22]
L34	CS1	literature	Kirdyanov, et al. [23]
L35	TYPA	literature	Benkova, et al. [24]
L36	TURA1	literature	Kujansuu, et al. [25]
L37	TURA2	literature	Fonti, et al. [26]
L38	TURA3	literature	Sidorova, et al. [27]
L39	UM1	literature	Tei, et al. [28]
L40	KOZ	literature	Takahashi, et al. [29]
L41	LENA	literature	MacDonald, et al. [30]
L42	MHXC	literature	Yasmeen, et al. [31]
L43	WEXC	literature	Yasmeen, et al. [31]
L44	TLHXC	literature	Yasmeen, et al. [31]
L45	BYH	literature	Zhang, et al. [32]
L46	QMG	literature	Zhang, et al. [32]
L47	WULAGM	literature	Wu, et al. [5]

References

1 Crowther, T. W. *et al.* Mapping tree density at a global scale. *Nature* **525**, 201-205, doi:10.1038/nature14967 (2015).

2 van de Pol, M. *et al.* Identifying the best climatic predictors in ecology and evolution. *Methods in Ecology and Evolution* **7**, 1246-1257, doi:10.1111/2041-210X.12590 (2016).

3 Frich, P. Observed coherent changes in climatic extremes during the second half of the twentieth century. *Climate Research* **19**, 193-212, doi:10.3354/cr019193 (2002).

4 Seftigen, K., Frank, D. C., Björklund, J., Babst, F. & Poulter, B. The climatic drivers of normalized difference vegetation index and tree-ring-based estimates of forest productivity are spatially coherent but temporally decoupled in Northern Hemispheric forests. *Global Ecology and Biogeography* **27**, 1352-1365, doi:10.1111/geb.12802 (2018).

5 Wu, X. *et al.* Growth decline linked to warming-induced water limitation in hemi-boreal forests. *Plos One* **7**, e42619, doi:10.1371/journal.pone.0042619 (2012).

6 Wu, X. *et al.* Stand-total tree-ring measurements and forest inventory documented climate-induced forest dynamics in the semi-arid Altai Mountains. *Ecological Indicators* **36**, 231-241, doi:10.1016/j.ecolind.2013.07.005 (2014).

7 Tei, S. *et al.* Strong and stable relationships between tree-ring parameters and forest-level carbon fluxes in a Siberian larch forest. *Polar Science* **21**, 146-157, doi:10.1016/j.polar.2019.02.001 (2019).

8 Belokopytova, L. V., Babushkina, E. A., Zhirnova, D. F., Panyushkina, I. P. & Vaganov, E. A. Climatic response of conifer radial growth in forest-steppes of south Siberia: Comparison of three approaches. *Contemporary Problems of Ecology* **11**, 366-376, doi:10.1134/S1995425518040030 (2018).

9 Babushkina, E. A. *et al.* Response of four tree species to changing climate in a moisture-limited area of south Siberia. *Forests* **10**, 999, doi:10.3390/f10110999 (2019).

10 Babushkina, E. A. *et al.* Prospects of using tree-ring earlywood and latewood width for reconstruction of crops yield on example of south Siberia. *Forests* **12**, 174, doi:10.3390/f12020174 (2021).

11 Knorre, A. A. *et al.* Twentieth century trends in tree ring stable isotopes (δ^{13} and δ^{18}O) of *Larix sibirica* under dry conditions in the forest steppe in Siberia. *Journal of Geophysical Research: Biogeosciences* **115**, doi:10.1029/2009JG000930 (2010).

12 Babushkina, E. A. & Belokopytova, L. V. Climatic signal in radial increment of conifers in forest-steppe of southern Siberia and its dependence on local growing conditions. *Russian Journal of Ecology* **45**, 325-332, doi:10.1134/S1067413614050038 (2014).

13 Chen, F. *et al.* A 225-year long drought reconstruction for east Xinjiang based on Siberia

larch (*Larix sibirica*) tree-ring widths: Reveals the recent dry trend of the eastern end of Tien Shan. *Quaternary International* **358**, 42-47, doi:10.1016/j.quaint.2014.11.055 (2015).

14 Gradel, A., Ganbaatar, B., Nadaldorj, O., Dovdondemberel, B. & Kusbach, A. Climate-growth relationships and pointer year analysis of a Siberian larch (*Larix sibirica* Ledeb.) chronology from the Mongolian mountain forest steppe compared to white birch (*Betula platyphylla* Sukaczev). *Forest Ecosystems* **4**, 22, doi:10.1186/s40663-017-0110-2 (2017).

15 Khansaritoreh, E. *et al.* Age structure and trends in annual stem increment of Larix sibirica in two neighboring Mongolian forest–steppe regions differing in land use history. *Trees* **31**, 1973-1986, doi:10.1007/s00468-017-1601-z (2017).

16 Dulamsuren, C., Hauck, M. & Leuschner, C. Recent drought stress leads to growth reductions in *Larix sibirica* in the western Khentey, Mongolia. *Global Change Biology* **16**, 3024-3035, doi:10.1111/j.1365-2486.2009.02147.x (2010).

17 Kukarskih, V. V., Devi, N. M., Moiseev, P. A., Grigoriev, A. A. & Bubnov, M. O. Latitudinal and temporal shifts in the radial growth-climate response of Siberian larch in the Polar Urals. *Journal of Mountain Science* **15**, 722-729, doi:10.1007/s11629-017-4755-7 (2018).

18 Demakov, I. P. Radial growth patterns of Siberian larch in plantations of the Republic Mari El. *IOP Conference Series: Earth and Environmental Science* **932**, 012010, doi:10.1088/1755-1315/932/1/012010 (2021).

19 Khishigjargal, M., Dulamsuren, C., Leuschner, H. H., Leuschner, C. & Hauck, M. Climate effects on inter- and intra-annual larch stemwood anomalies in the Mongolian forest-steppe. *Acta Oecologica* **55**, 113-121, doi:10.1016/j.actao.2013.12.003 (2014).

20 Nikolaev, A. N., Fedorov, P. P. & Desyatkin, A. R. Influence of climate and soil hydrothermal regime on radial growth of *Larix cajanderi* and *Pinus sylvestris* in Central Yakutia, Russia. *Scandinavian Journal of Forest Research* **24**, 217-226, doi:10.1080/02827580902971181 (2009).

21 Arzac, A. *et al.* Increasing radial and latewood growth rates of *Larix cajanderi* Mayr. and *Pinus sylvestris* L. in the continuous permafrost zone in Central Yakutia (Russia). *Annals of Forest Science* **76**, 96, doi:10.1007/s13595-019-0881-4 (2019).

22 Kharuk, V. I., Ranson, K. J., Im, S. T. & Petrov, I. y. A. Climate-induced larch growth response within the central Siberian permafrost zone. *Environmental Research Letters* **10**, 125009, doi:10.1088/1748-9326/10/12/125009 (2015).

23 Kirdyanov, A. V. *et al.* Long-term ecological consequences of forest fires in the continuous permafrost zone of Siberia. *Environmental Research Letters* **15**, 034061, doi:10.1088/1748-9326/ab7469 (2020).

24 Benkova, A. V., Mashukov, D. A., Benkova, V. E., Prokushkin, A. S. & Shashkin, A. V. The effect of slope exposition on the growth dynamics of *Larix gmelinii* in permafrost conditions of Central Siberia. I. Differences in tree radial dynamics growth in the north- and south-facing slopes (in Russian). *Sibirskij Lesnoj Žurnal* **2**, doi:10.15372/SJFS20150402 (2015).

25 Kujansuu, J. *et al.* Climatic responses of tree-ring widths of *Larix gmelinii* on contrasting north-facing and south-facing slopes in central Siberia. *Journal of Wood Science* **53**, 87-93, doi:10.1007/s10086-006-0837-9 (2007).

26 Fonti, M. V. *et al.* Long-term variability of anatomic features of annual tree rings of larch, pine and spruce in the permafrost zone in central Siberia. *Contemporary Problems of Ecology* **12**, 692-702, doi:10.1134/S1995425519070035 (2019).

27 Sidorova, O. V. *et al.* Do centennial tree-ring and stable isotope trends of *Larix gmelinii* (Rupr.) Rupr. indicate increasing water shortage in the Siberian north? *Oecologia* **161**, 825-835, doi:10.1007/s00442-009-1411-0 (2009).

28 Tei, S., Yonenobu, H., Sugimoto, A., Ohta, T. & Maximov, T. C. Reconstructed summer Palmer Drought Severity Index since 1850 AD based on $\delta^{13}C$ of larch tree rings in eastern Siberia. *Journal of Hydrology* **529**, 442-448, doi:10.1016/j.jhydrol.2015.01.085 (2015).

29 Takahashi, K., Homma, K., Shiraiwa, T., Vetrova, P. V. & Hara, T. Climatic factors affecting the growth of *Larix cajanderi* in the Kamchatka Peninsula, Russia. *Eurasian Journal of Forest Research* **3**, 1-9 (2001).

30 MacDonald, G. M., Case, R. A. & Szeicz, J. M. A 538-year record of climate and treeline dynamics from the lower lena river region of northern Siberia, Russia. *Arctic and Alpine Research* **30**, 334-339, doi:10.1080/00040851.1998.12002908 (1998).

31 Yasmeen, S. *et al.* Contrasting climate-growth relationship between *Larix gmelinii* and *Pinus sylvestris* var. *mongolica* along a latitudinal gradient in Daxing'an Mountains, China. *Dendrochronologia* **58**, 125645, doi:10.1016/j.dendro.2019.125645 (2019).

32 Zhang, T., Yu, S., Yuan, Y., Huang, L. & Jiang, S. Development of a *Larix principis-rupprechtii* tree-ring width chronology and its climatic signals for the southern Greater Higgnan Mountains. *Geochronometria* **45**, 1-9, doi:10.1515/geochr-2015-0082 (2018).
