# Supplementary material for: Reassessment of growth-climate relations indicates the potential for decline across Eurasian boreal larch forests
Source: Nat Commun. 2023 Jun 8;14:3358. doi: 10.1038/s41467-023-39057-5 (PMC10250375; doi:10.1038/s41467-023-39057-5)
Supplement: Supplementary file 3 — Description of Additional Supplementary Files [file 41467_2023_39057_MOESM3_ESM.pdf]

Description of Additional Supplementary Files

File Name: Supplementary Data 1

Description: Detailed information of sampling populations.

File Name: Supplementary Data 2

Description: Detailed information of meteorological stations.
