# Supplementary material for: Reassessment of growth-climate relations indicates the potential for decline across Eurasian boreal larch forests
Source: Nat Commun. 2023 Jun 8;14:3358. doi: 10.1038/s41467-023-39057-5 (PMC10250375; doi:10.1038/s41467-023-39057-5)
Supplement: Supplementary file 6 — Reporting Summary [file 41467_2023_39057_MOESM6_ESM.pdf]

nature portfolio

Corresponding author(s): Yuan Jiang

Last updated by author(s): May 22, 2023

Reporting Summary

Nature Portfolio wishes to improve the reproducibility of the work that we publish. This form provides structure for consistency and transparency in reporting. For further information on Nature Portfolio policies, see our Editorial Policies and the Editorial Policy Checklist.

Statistics

For all statistical analyses, confirm that the following items are present in the figure legend, table legend, main text, or Methods section.

n/a	Confirmed	
☐	☒	The exact sample size (*n*) for each experimental group/condition, given as a discrete number and unit of measurement
☐	☒	A statement on whether measurements were taken from distinct samples or whether the same sample was measured repeatedly
☐	☒	The statistical test(s) used AND whether they are one- or two-sided<br>*Only common tests should be described solely by name; describe more complex techniques in the Methods section.*
☐	☒	A description of all covariates tested
☐	☒	A description of any assumptions or corrections, such as tests of normality and adjustment for multiple comparisons
☐	☒	A full description of the statistical parameters including central tendency (e.g. means) or other basic estimates (e.g. regression coefficient) AND variation (e.g. standard deviation) or associated estimates of uncertainty (e.g. confidence intervals)
☐	☒	For null hypothesis testing, the test statistic (e.g. *F*, *t*, *r*) with confidence intervals, effect sizes, degrees of freedom and *P* value noted<br>*Give P values as exact values whenever suitable.*
☒	☐	For Bayesian analysis, information on the choice of priors and Markov chain Monte Carlo settings
☒	☐	For hierarchical and complex designs, identification of the appropriate level for tests and full reporting of outcomes
☐	☒	Estimates of effect sizes (e.g. Cohen's *d*, Pearson's *r*), indicating how they were calculated

Our web collection on statistics for biologists contains articles on many of the points above.

Software and code

Policy information about availability of computer code

Data collection	The data was collected directly, or available sources, listed in the manuscript.
Data analysis	Data analyses and result visualization in this study were performed in R (version 4.1.3). R packages used: caret (v6.0-93), data.table (v1.14.6), dplR (v1.7.4), dplyr (v1.0.10), ggplot2 (v3.4.0.9000), ggpubr (v0.5.0), gtools (v3.9.4), maptools (v1.1-6), raster (v3.6-13), and rgdal (v1.6-4).The map was drawn by ArcGIS 10.2 for Desktop (ESRI, Inc.).

For manuscripts utilizing custom algorithms or software that are central to the research but not yet described in published literature, software must be made available to editors and reviewers. We strongly encourage code deposition in a community repository (e.g. GitHub). See the Nature Portfolio guidelines for submitting code & software for further information.

Data

Policy information about availability of data

All manuscripts must include a data availability statement. This statement should provide the following information, where applicable:

- Accession codes, unique identifiers, or web links for publicly available datasets
- A description of any restrictions on data availability
- For clinical datasets or third party data, please ensure that the statement adheres to our policy

In the method section each data source is listed.
The ITRDB tree-ring width data was obtained from https://www.ncei.noaa.gov/products/paleoclimatology/tree-ring. The ITRDB data we used, the tree-ring

chronologies we digitized from previously-published literatures, and the tree-ring data we directly collected have been uploaded to the repository, available via https://doi.org/10.6084/m9.figshare.22590235.
The Worldclim datasets were obtained from https://www.worldclim.org. The CRU TS 4.05 dataset was obtained from https://www.uea.ac.uk/groups-and-centres/climatic-research-unit. The daily mean temperature records from the meteorological stations operated by the Global Historical Climatology Network and the China Meteorological Data Service Center were obtained from https://www.ncei.noaa.gov/cdo-web/search?datasetid=GHCND and http://data.cma.cn, respectively, and have also been uploaded to the repository together with the tree-ring data.

Research involving human participants, their data, or biological material

Policy information about studies with human participants or human data. See also policy information about sex, gender (identity/presentation), and sexual orientation and race, ethnicity and racism.

Reporting on sex and gender	n/a
Reporting on race, ethnicity, or other socially relevant groupings	n/a
Population characteristics	n/a
Recruitment	n/a
Ethics oversight	n/a

Note that full information on the approval of the study protocol must also be provided in the manuscript.

Field-specific reporting

Please select the one below that is the best fit for your research. If you are not sure, read the appropriate sections before making your selection.

☐ Life sciences ☐ Behavioural & social sciences ☒ Ecological, evolutionary & environmental sciences

For a reference copy of the document with all sections, see nature.com/documents/nr-reporting-summary-flat.pdf

Ecological, evolutionary & environmental sciences study design

All studies must disclose on these points even when the disclosure is negative.

Study description	We focus on the extensive larch forests dominant in boreal Eurasia with great ecological relevance, faster warming rates, but less scientific attention compared with boreal evergreen conifers in western Europe and North America.<br>Correlating tree-ring data with rigid calendar-based temperature series has detected universal growth responses almost exclusively in the margins of boreal Eurasia. We infer that the deciduous nature of larch, together with the short and highly variable boreal growing seasons, are likely to dilute the footprints of climate variations in radial growth, resulting in inefficiency of calendar-based approach. Therefore, we developed a novel method to construct temporally-flexible and physiologically-informed temperature series to assess growth-temperature relations of Eurasian boreal larch forests using an extensive tree-ring network composed of 8,544 annual radial growth series of 5,089 larch trees and 260 larch populations covering the distributions of boreal larch.<br>We detected widespread and spatially heterogeneous growth-temperature responses across Eurasian boreal larch forests and identified local climate as the main driver of this heterogeneity. We then quantified the driving relationship into logistic models to estimate the probability of showing negative growth-temperature responses across larch distribution and build climate boundaries that well discriminated between populations showing different response patterns. We further projected the future dynamics of different response patterns across larch distribution throughout the 21st century by applying the models to climate projections from 25 GCMs under four SSPs, showing emerging negative effects and diminishing benefits from warming.<br>Our findings reminded the potentially overlooked and presumably increasing risks of warming to Eurasian boreal forests, warning against blind optimism and potential wasted management opportunities in the context of rapid warming.
Research sample	Tree-ring width series from 131 Siberian larch (Larix sibirica) populations and 129 Dahurian larch (Larix gmelinii) populations were used to represent the two dominant larch species in boreal Eurasia, collected from three sources: (i) the International Tree Ring Data Bank (ITRDB), 193 populations (7,271 raw tree-ring width series from 4,605 trees), 27 of which were contributed by us; (ii) field work during the 2010s, three Siberian larch populations and 17 Dahurian larch populations (1,273 raw tree-ring width series from 484 trees) from the Altai Mountains in northwest China and the Greater Khingan Range in northeast China, respectively, with the aim of expanding the data coverage to include the southernmost distributions of the two species; and (iii) previously-published research, specifically 47 population tree-ring chronologies (established based on 2,785 core samples from 1826 trees) digitalized from published literature for further spatially enriching our network. ITRDB is the largest tree-ring data collection available. Sampling, processing procedure, and measurement of tree-ring width data from all sources followed the basic principles and standard methods of dendrochronology, ensuring consistency in data standards.
Sampling strategy	The sampling of tree-ring data in our field work followed the principles of replication in dendrochronology. We selected the sampling populations avoiding special terrain, special habitat microenvironments, human interferences (forestry or agricultural activities), and extreme natural disturbances (wildfires or pest outbreaks). 25-50 mature trees were randomly selected in each population and cored 2-4 times per tree at breast height using increment borers.

Data collection	The ITRDB tree-ring width data were downloaded from https://www.ncei.noaa.gov/products/paleoclimatology/tree-ring. We downloaded all available data of these two larch species in the ITRDB as of July 2022. Tree-ring cores obtained from our field work were collected, cross-dated, and measured following standard sampling and processing dendrochronological methodologies. Wenqing Li, Yuan Jiang, Neil Pederson, Enzai Du, Shoudong Zhao, Manyu Dong, and Fang Wu conducted field work and sample processing. Previously-published tree-ring chronologies were digitalized from literature providing accurate geographic coordinates and suitable temporal coverage, which were searched via the Web of Science (http://isiknowledge.com) and Google Scholar (https://scholar.google.com). Wenqing Li and Yuan Jiang completed the digitization of the published chronologies.<br>WorldClim datasets (gridded climate layers of mean annual temperature and mean annual precipitation over 1960-1990 and 1970-2000; downscaled GMIP6 gridded climate projections from 25 general circulation models over four periods under four SSPs) were downloaded from https://www.worldclim.org. Daily mean temperature records from the meteorological stations were downloaded from https://www.ncei.noaa.gov/cdo-web/search?datasetid=GHCND and http://data.cma.cn.
Timing and spatial scale	Tree-ring width data are annual radial growth measurements. Although the time span of each tree-ring width series depends on location and source, we used the tree-ring data and climate data during 1960-1990 and 1970-2000 in parallel for analysis.<br>The tree-ring network we compiled covers the distribution of Eurasian boreal larch forests, consisting of 8,544 annual radial growth series of 5,089 larch trees and 260 larch populations, from 43°15'N to 72°30'N in latitude and from 29°34'E to 169°00'E in longitude.
Data exclusions	Series available in the ITRDB for the region but that did not fully cover the target time window of our analyses (2,854 tree-ring width series), were not used. In total, 8,544 raw ring-width series were used.
Reproducibility	The R code of T-linked method and other custom codes used, as well as the necessary data for this study, are provided for reproducibility, available via https://doi.org/10.6084/m9.figshare.22590235.
Randomization	Our study does not include control/treatment differences. Used tree-ring data are from observational studies under natural conditions not manipulative experiments. All available growth data were considered.
Blinding	Our study does not include control/treatment sample assignment. Used tree-ring data are from observational studies under natural conditions not manipulative experiments.

Did the study involve field work? ☒ Yes ☐ No

Field work, collection and transport

Field conditions	We conducted field work to collect tree-ring cores from three Siberian larch populations in the Altai Mountains in northwest China, and 17 Dahurian larch populations in the Greater Khingan Range in northeast China. For the Siberian larch populations, the climate is arid and semi-arid temperate continental, with 1970-2000 mean annual temperatures of -2.6-0.0 °C and mean annual precipitations of 300-340 mm (WorldClim version 2.1). For the Dahurian larch populations, the climate is semi-humid temperate monsoon, with 1970-2000 mean annual temperatures of -5.0-1.0 °C and mean annual precipitations of 450-521 mm.
Location	Location of the sampling populations in the Altai Mountains in northwest China: 48°22'48"-48°30'10"N latitude, 86°23'55"-87°10'50"E longitude, and 1477-2069 m a.s.l. elevation.<br>Location of the sampling populations in the Greater Khingan Range in northeast China: 47°09'36"-53°15'00"N latitude, 120°24'09"-124°24'15"E longitude, 542-1330 m a.s.l. elevation.<br>The specific longitude, latitude, and elevation of each sampling population are listed in Appendix A.
Access & import/export	Site access and sampling were conducted under the guidelines and assistance of local forestry management departments, in full compliance with relevant laws and regulations.
Disturbance	We adhered to “leave no trace principles” when conducting field work to minimize the disturbance to the forests from which we collected tree-ring cores.

Reporting for specific materials, systems and methods

We require information from authors about some types of materials, experimental systems and methods used in many studies. Here, indicate whether each material, system or method listed is relevant to your study. If you are not sure if a list item applies to your research, read the appropriate section before selecting a response.

Materials & experimental systems

n/a	Involved in the study
☒	☐ Antibodies
☒	☐ Eukaryotic cell lines
☒	☐ Palaeontology and archaeology
☒	☐ Animals and other organisms
☒	☐ Clinical data
☒	☐ Dual use research of concern
☒	☐ Plants

Methods

n/a	Involved in the study
☒	☐ ChIP-seq
☒	☐ Flow cytometry
☒	☐ MRI-based neuroimaging
